# A Phase 2A randomized, double-blind, placebo-controlled pilot trial of GM604 in patients with Amyotrophic Lateral Sclerosis (ALS Protocol GALS-001) and a single compassionate patient treatment (Protocol GALS-C)

**DOI:** 10.12688/f1000research.10519.1

**Published:** 2017-03-07

**Authors:** Mark Kindy, Paul Lupinacci, Raymond Chau, Tony Shum, Dorothy Ko

**Affiliations:** 1 Department of Pharmaceutical Sciences, College of Pharmacy, University of South Florida, Tampa, FL, USA; 2 Department of Mathematics and Statistics, Villanova University, Villanova, PA, USA; 3 Genervon Pharmaceuticals LLC, Pasadena, CA, USA

**Keywords:** ALS, ALSFRS-R, FVC, ALS Biomarkers, neurodegeneration, signaling

## Abstract

**Background**

Amyotrophic lateral sclerosis (ALS) is a fatal neurodegenerative disease that lacks effective treatment options. Genervon has discovered and developed GM604 (GM6) as a potential ALS therapy. GM6 has been modeled upon an insulin receptor tyrosine kinase binding motoneuronotrophic factor within the developing central nervous system.

**Methods**

This was a 2-center phase 2A, randomized, double-blind, placebo-controlled pilot trial with 12 definite ALS patients diagnosed within 2 years of disease onset. Patients received 6 doses of GM604 or placebo, administered as slow IV bolus injections (3x/week, 2 consecutive weeks). Objectives were to assess the safety and efficacy of GM604 based on ALSFRS-R, FVC and selected biomarkers (TDP-43, Tau and SOD1, pNFH). This report also includes results of compassionate treatment protocol GALS-C for an advanced ALS patient.

**Results**

Definite ALS patients were randomized to one of two treatment groups (GM604, n = 8; placebo, n = 4). 2 of 8 GM604-treated patients exhibited mild rash, but otherwise adverse event frequency was similar in treated and placebo groups. GM604 slowed functional decline (ALSFRS-R) when compared to a historical control (P = 0.005). At one study site, a statistically significant difference between treatment and control groups was found when comparing changes in respiratory function (FVC) between baseline and week 12 (P = 0.027). GM604 decreased plasma levels of key ALS biomarkers relative to the placebo group (TDP-43, P = 0.008; Tau, P = 0.037; SOD1, P = 0.009). The advanced ALS patient in compassionate treatment demonstrated improved speech, oral fluid consumption, mouth suction with GM604 treatment and biomarker improvements.

**Conclusions**

We observed favorable shifts in ALS biomarkers and improved functional measures during the Phase 2A study as well as in an advanced ALS patient. Although a larger trial is needed to confirm these findings, the present data are encouraging and support GM604 as an ALS drug candidate.

## Introduction

Amyotrophic lateral sclerosis (ALS) is a devastating disease for which no effective treatment has been discovered ^1^. During the last twenty years, dozens of ALS drug candidates have been tested but have unfortunately failed during clinical trials ^2^. This astounding record of uniform failure may be attributed to the fact that the classic drug development model – which aims to design single-target drugs – is simply inadequate for rapid, complex and multifactorial diseases like ALS ^3^.

Genervon decided to look for and discovered endogenous regulators of the developing nervous system, and hypothesized that such regulators may have the capacity to monitor and repair neurological diseases ^4, 5^. Genervon’s approach was to base drug design on these regulatory proteins, leading to development of GM604 (GM6) ^6^. GM604 is a peptide with a sequence identical to one of the active sites of human motoneuronotrophic factor (MNTF) ^7^. MNTF is an endogenous human embryonic stage neural regulatory and signaling peptide that controls the development, monitoring and correction of the human nervous system ^4, 5^. This activity of MNTF is replicated by GM604 to provide a potent disease-modifying drug candidate that modulates many processes including inflammation, apoptosis, and hypoxia ^4, 5, 7^. In pre-clinical studies, we have shown that GM604 acts as a neuro-protective agent in animal models of neurological disease ^7^. In these studies, GM604 was found to promote neuroprotection, neurogenesis, neural development, neuronal signaling, neural transport, and other processes ^4– 7^. Recently, we have demonstrated that GM604 modulates many ALS-associated genes, promoting decreased expression of superoxide dismutase (SOD1), repression of genes associated with the intrinsic apoptosis pathway, and increased expression of genes associated with mitosis and cell division ^8^.

This paper reports findings from a multi-center Phase 2A, double-blind, randomized, placebo-controlled pilot trial in 12 patients with Familial or Sporadic ALS diagnosed as definite ALS according to the El Escorial Criteria ^9, 10^. Objectives of the trial were to assess proof of principle; i.e., to determine whether a 2-week IV bolus treatment with GM604 can (i) be safely used and tolerated without significant adverse effects, (ii) favorably alter ALS biomarkers, and (iii) delay progression based upon key clinical indices. This report also includes results of protocol GALS-C for an advanced ALS patient who has been quadriplegic and on a ventilator since 2008 (IND number 120052).

## Methods

This was a multi-center Phase 2A, double-blind, randomized, placebo-controlled pilot trial in 12 patients with Familial or Sporadic ALS. Objectives were to test the safety, tolerability and efficacy of GM604 and to assess changes in clinical disease progression and selected ALS biomarkers. GM604 has received Orphan Drug Designation 14-4247 by the FDA Office of Orphan Products Development for treatment of ALS and Orphan Designation (EU/3/16/1662) from the European Medical Commission. Genervon received Fast Track Designation for GM604 to treat ALS (IND number 118,420) by FDA Office of Drug Evaluation I, CDER. Genervon also received Fast Track designation for GM604 to treat Ischemic Stroke (IND number 77,789).

This report also includes results of protocol GALS-C for an advanced ALS patient who has been quadriplegic and on a ventilator since 2008 (IND number 120052). It is an Expanded Access Use applied by a physician to treat his/her individual patient. The physician submits a new IND request with Form 1571 to FDA including treatment protocol, CV, IRB approval, Informed Consent Form, Medical License etc. and a Letter of Authorization (LOA) signed by the sponsor to refer to the sponsor’s IND for information regarding the investigational drug in Investigator’s Brochure, Chemistry, Manufacturing and Controls (CMC) information, and pharmacology and toxicology. After FDA approves the Expanded Access Treatment request by the physician, an IND number 120052 is assigned for the Expanded Access Use for the GALS-C patient treatment with GM60404. GM60404 is only shipped to the physician after the physician received FDA’s Study May Proceed letter. All components required by FDA are fulfilled before FDA will assign an IND number and allow the treatment to proceed. Since GALS-C is not a clinical trial, it is not registered with clinicaltrials.gov. FDA now has a simpler form for Individual Patient Expanded Access Applications (FDA Form 3926).

### Randomization

Patients who qualified for the study were enrolled and assigned a unique patient number. The patient’s initials and identification number were written on all source documents. Only the site number and patient’s study ID number were written on CRF pages, documents sent to central readers, and CSF and blood samples sent to central lab for processing.

Patients fulfilling the eligibility criteria were assigned randomization codes, starting with number 0101, with 0100 series for Site 001 and 0200 series for Site 2. The patient number was assigned in sequential order as the patient enrolled. 6 patients were enrolled at each site. 8 patients were randomized to receive GM604 and 4 patients were randomized to receive placebo control. The statistical analysis team generated a list of randomization code and sent the list to the pharmacist of each site. The study site pharmacist retained the original treatment randomization schedule in a secure location. All activities of this study were conducted in a double-blinded, randomized, placebo controlled manner.

### Ethics statement

The Phase 2A study was performed in compliance with the current International Conference on Harmonization (ICH) Good Clinical Practice (GCP) guidance and the current version of the Declaration of Helsinki of the World Medical Association ^11^. The final protocol and informed consent form were reviewed and approved by the Columbia University Institutional Review Board (CU IRB) for Site 001 (Columbia University Medical Center) and by the Partners Human Research Committee (PHRC) for Site 002 (Massachusetts General Hospital). All patients who participated were fully informed about the study in accordance with GCP guidelines, federal regulations, HIPAA, and local requirements ^12^. The trial was posted on clinicaltrials.gov on May 8, 2013. (NCT01854294) ^13^. The GALS-C is not a clinical trial but an Expanded Access for compassionate treatment. IRB approval was received from Bay Area Regional IRB of Dignity Health.

### ALS Protocol GALS-001

***Subject Population***. There are a total of two study sites: Columbia University Medical Center, New York and Massachusetts General Hospital. Definite ALS patients were randomized at each site to four GM604 treated and two placebo treated. Eligible patients met the El Escorial criteria for ALS ^9, 10^. At screening, symptom onset had occurred within the previous 24 months and forced vital capacity (FVC) was ≥65% of predicted capacity based upon age, height, and gender. Mean disease duration was 8.15 months, ranging from 2.7 to 16.5 months across treatment groups. Patients in the placebo group reported a slightly longer duration of disease, with a median duration of 8.90 months, compared with a median of 5.24 months for patients in the GM604 treatment group. The demographic profile of the placebo and GM604 treatment groups was matched in terms of age, with medians of 54.5 and 56.0 years in the placebo and treatment groups, respectively. The mean age of patients was 55.7 years, ranging from 45 to 68 across treatment groups. The majority of patients (66.7%; 8/12) were male. Gender distribution was slightly different in the two treatment groups, with an equal number of males and females in the placebo group (2/2) and a majority of males in the GM604 treatment group (75%; 6/8). All 4 of the females were at least 2 years post-menopausal. All 12 patients were Caucasian.

Patients were excluded if they had a bleeding disorder, allergy to local anesthetics, or medical or surgical conditions in which lumbar puncture was contraindicated, e.g., elevated cerebrospinal fluid (CSF) pressure. Prohibited medications included anti-platelet or anticoagulant drugs such as Plavix, non-steroidal anti-inflammatory drugs (NSAIDs), ticlid, warfarin or coumadin. Patients may have been on a stable dose of riluzole for at least a month before screening, but riluzole was not initiated during the trial. We note that some biomarker data were missing due to hemolysis of samples, technical issues, or patients who missed clinical appointments for sample collection. These missing data were excluded from analyses.

***Procedures***. Following screening, patients were randomized to receive GM604 (n=8) or placebo (n=4). Patients received 6 doses of 320 mg GM604 or placebo, administered as slow IV bolus injections on Monday, Wednesday, and Friday of weeks 1 and 2. Clinical assessments included the ALS Functional Rating Scale – Revised (ALSFRS-R) ^14^, FVC ^15– 17^, timed up & go (TUG) ^18^, and hand-held dynamometry (HHD) ^19^. Assessements were conducted at screening, before the first dose (baseline), after the last (6 ^th^) dose at week 2, and at weeks 6 and 12. Safety and tolerability were evaluated based on the frequency of adverse events, vital signs, electrocardiography (ECG) measurements, physical and neurological examinations, safety laboratory monitoring, and hypersensitivity and injection site reactions ^20^. The following visit windows were allowed: visits 1 (baseline and first dosing) to 6 (last dosing, 2 weeks): ± 1 day; visit 7 (4 weeks after last dosing, 6 weeks total): ± 7 days; visit 8 (10 weeks after last dosing, 12 weeks total): ± 14 days. We note that one patient (in the GM604 treatment group) returned to Germany where he resides and did not return for the week 12 assessment, although he did contact investigators to provide ALSFRS-R by phone. A total of 11 patients thus received all 6 doses of the study drug, with one patient receiving 5 doses of the drug.

***Biomarkers***. The biomarkers SOD1, phosphorylated neurofilament heavy chain (pNFH) ^21^, total tau, and TDP-43 were assessed at baseline, after the initial week 2 (4 ^th^) dose (plasma only), after the last (6 ^th^) dose (also in week 2) and at weeks 6 and 12. TDP-43 (TAR DNA-binding protein 43, transactive response DNA binding protein 43 kDa) is a protein encoded by the *TARDBP* gene. Mutations in the *TARDBP* gene are associated with neurodegenerative disorders including ALS ^20, 22– 25^. We note that some biomarker data were missing due to technical issues with sample processing, or patients who missed clinical appointments for sample collection. These missing data were excluded from analyses.

***Efficacy assessments***. The ALSFRS-R is used to assess disability in ALS patients. It is a total score derived from sub-scores in the following categories: speech, salivation, swallowing, handwriting, cutting food, dressing and hygiene, turning in bed, walking, climbing stairs, dyspnea, orthopnea, and respiratory insufficiency. The score decreases as the disease progresses ^14^.

The FVC, measured as a percentage, is used to assess respiratory function and is an indicator of disease progression. FVC also decreases with disease progression ^15– 17^.

TUG is used to predict falls in ALS. In this study, TUG in ambulatory participants with no assistance was measured and recorded with videotaping ^18^ The TUG was measured in seconds rounded to 1 decimal place, with smaller estimates indicating that a patient can walk faster. As ALS progresses, however, the walking pace may slow, or the patient may be unable to perform TUG. In the present study, TUG performed with assistance was excluded and treated as missing data.

HHD is used to measure muscle strength. HHD measures are dependent on the ability of the evaluator to overpower the subject's strength ^19^. In this study, the clinician stabilized the limb segment while encouraging the patient to exert as much force as possible against an isometric HHD, and the maximum force was recorded by the HHD. Each site was tested in duplicate (triplicate if the first 2 results were more than 15% apart) and the result was measured in pounds using 1 decimal. The average of replicates for each clinical site was calculated and used in the analysis for each of the time points. ****

***Statistical Analyses***. The percentage change from baseline of each biomarker in plasma and CSF was compared between treatments using a 2-sample t-test and Wilcoxon Rank Sum test. Progressive changes in clinical endpoints were examined using mixed effects modeling (ALSFRS-R, FVC, TUG, grip strength and HHD scores). Rates of disease progression were compared between GM604- and placebo-treated patients. Additionally, we made comparisons to placebo-treated patients from the Northeast ALS Consortium (NEALS) database showing stable rates of decline ( https://www.alsconsortium.org/).

## Results

### ALS Protocol GALS-001

Study Initiation date was 16 May 2013 (first Subject pre-screened0, 03 September 2013 (first Subject screened), Study completion/Termination Date (last Subject completed) was 11 April 2014.

***Safety***. Of 12 patients enrolled in the study, 9 reported at least one adverse event. Overall, in the GM604 treatment group, 5 of 8 patients experienced at least one treatment emergent adverse event (TEAE) and 4 of 4 patients in the placebo group experienced at least 1 TEAE. No unexpected findings were observed. Consistent with protocol-defined expected adverse reactions, the most frequently reported AEs by GM604-treated patients in the present study were falls (4 patients, 50%), puncture site pain (3 patients, 37.5%), rash (2 patients) and headache (2 patients, 25%). Of these most commonly reported TEAEs in GM604-treated patients, falls (1 patient, 25%), puncture site pain (1 patient, 25%) and headache (2 patients, 50%) were reported in placebo-treated patients.

Adverse events in the ‘general disorders and administration site conditions’ system organ class (SOC) were the most frequently experienced adverse events (7 patients and 61 total events in both the GM604 and placebo-treated groups). A serious adverse event that required inpatient hospitalization, shortness of breath 24 days after the first dose of GM604 (12 days after the last dose), was experienced by one patient in the GM604 treatment group. This patient received the full 6 doses of GM604 treatment and then left the study site and flew back to Germany. There was no additional GM604 administered to this patient during the hospital stay in Germany that could have affected the outcome of the results.

It was determined by investigators that this serious adverse event was most likely due to the natural progression of ALS and was thus unrelated to the investigational product. No deaths or withdrawals due to adverse events occurred.

There were no clinically meaningful differences noted between patients who received GM604 and those who received placebo for changes over time in clinical laboratory tests, hematology parameters, or urinalysis results. There were no clinically meaningful differences noted between patients who received GM604 and those who received placebo for changes over time in ECGs, vital signs, physical findings, neurological examination, or other observations related to safety.

Grade 1 hypersensitivity reactions were reported by one patient receiving placebo (visit 2 during week 1) and one patient receiving GM604 (visit 5 during week 2). All other patients reported an absence of hypersensitivity (Grade 0) reactions. There was no indication of QT prolongation as no patient receiving GM604 had a QT or QTcB (QT corrected using Bazett’s formula) result above 450 msec.

***Biomarker findings***. Previous clinical studies in patients with ALS have suggested that biomarker concentrations in plasma, serum, and CSF can be predictive of disease progression ^26– 33^. Therefore, a primary endpoint of the present study was to examine the percentage change of each biomarker between baseline and week 12.

**Figure 1.**
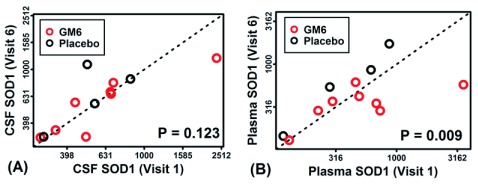
SOD1 protein in CSF and plasma of GM6- and placebo control-treated patients (GALS-001). SOD1 was measured in cerebrospinal fluid (CSF) and plasma at baseline (visit 1) and following 6 doses of GM6 over 2 weeks (visit 6). In ( **A**) and ( **B**) estimated SOD1 levels (pg/ml) are plotted (log _10_-transformed scale), with each point representing a single ALS patient. Patients below the diagonal showed decreased SOD1 post-treatment. P-values (lower right) were generated from the comparison of SOD1 measurements between visits 1 and 6 (p=0.009 one-tailed paired t-test performed using log10-transformed SOD1 estimates).

**Figure 2.**
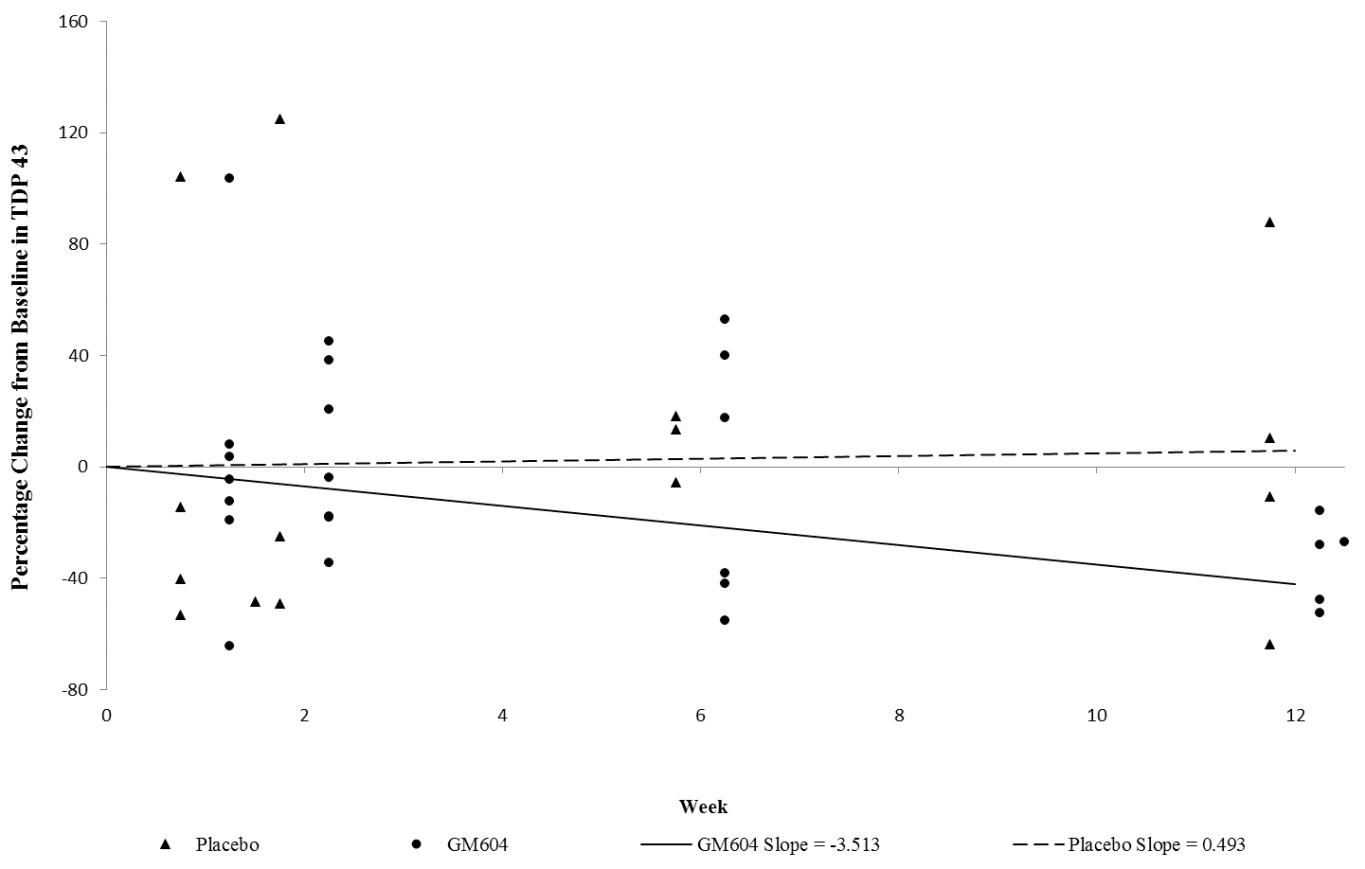
Percentage change in slope by treatment interaction in plasma TDP-43 over time, from baseline (visit 1) through to week 12 (visit 8) (GALS-001). The mean change in slope for plasma TDP-43 from baseline to week 12 in the GM6 treated groups was -3.513 pg/ml, which represents a decrease of 34%, while in the placebo group the mean change in slope was 0.493 pg/ml, which represents an increase of 6% from baseline. (p=0.0078, test for the significance of difference between the slopes, GM604 vs. placebo.) (To analyze disease progression, the results of the biomarker assays were analyzed using a mixed model repeated measures analysis. Commensurate with the design of the study, a mixed effects model was used to examine differences in the percentage change from baseline over time for each of the biomarkers. The unstructured covariance structure was used to model the intra-subject correlation. Since the percentage change from baseline at baseline is zero for all subjects, the y-intercept was removed from the model which will force the y-intercept to be 0. The explanatory variables that were added to the model include the week (2, 6, 12) as a numerical variable, treatment (GM604, placebo) and the treatment by week interaction. The model was run using all results through to week 6 and then again using all results through to week 12. The p-value indicates the significance of the difference in slopes between GM604 and placebo ( Dataset 15 ^39^) up to week 12.

**Figure 3.**
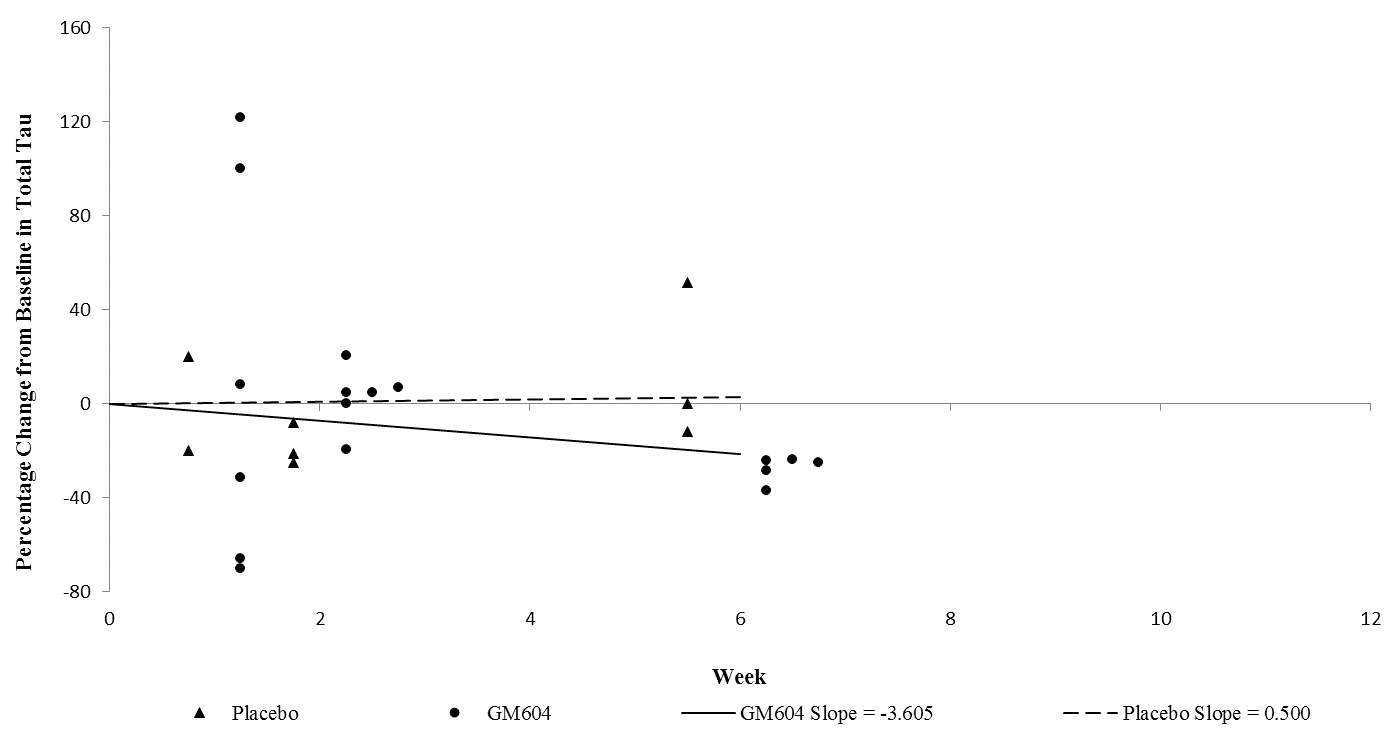
Percentage change in plasma total tau over time, from baseline (visit 1) through to week 6 (visit 7) (GALS-001). The mean percentage change from baseline for plasma total tau in GM6 treated patients was -27.69%, while the mean percentage change from baseline for the placebo group was 13.23% (p = 0.0369, Wilcoxon Rank Sum Test). This tests the significance of the difference in percentage change between the GM604 treated group and the placebo group from baseline to week 6 ( Dataset 14 ^52^).

In plasma samples, percentage change in plasma SOD1 at visit 6 (end of week 2) was lower than at baseline (p=0.0550, two sample t-test) following GM604 treatment compared with placebo which did not lower SOD1 ( Table 1, Figure 1, Dataset 1 ^34^ and Dataset 13 ^35^). Percentage change in plasma total tau was significantly decreased, approximately -28% below baseline (p=0.0369 95% CI, Wilcoxon Rank Sum test) at week 6 (visit 7) after active GM604 treatment compared to placebo ( Table 1, Figure 3, Dataset 2 ^36^ and Dataset 14 ^37^). Percentage change in slope by treatment interaction in plasma TDP-43 from baseline (visit 1) through to week 12 (visit 8) was -34% in the GM604 treated group and +6% in the placebo group (p=00078 95% CI). The p-value of 0.0078 indicates a significant difference in slopes between GM604 and placebo up to week 12 ( Table 1, Figure 2, Dataset 3 ^38^ and Dataset 15 ^39^).

**Table 1.** GALS-001 biomarker results. For Plasma SOD1 and Plasma Total tau: ^1^The P-value was obtained from a two-sample t-test for the difference in the change from baseline values between placebo and GM604. ^2^The P-value was obtained from a Wilcoxon Rank Sum Test for the difference in the change from baseline values between placebo and GM604. For Plasma TDP-43: Results were obtained from a mixed model repeated measures analysis for the change from baseline as the response variable with explanatory variables for week and the week by treatment interaction. The y-intercept was taken out of the model and forced to be 0 as the percentage change from baseline at baseline must be 0. The unstructured covariance structure was used to model the intra-subject correlation. ^3^The P-value indicates the significance of the difference in slopes between GM604 and Placebo.

Target biomarkers	Biomarkers	GM604 treated patients	Placebo patients	Comments
	Plasma SOD1	↓Reduced significantly at week 2 (p=0.055) ^1^	↑Increased	Significant reduction of SOD1 indicates it is a target of GM604
	CSF SOD1	↓Reduced	↑Increased	SOD 1 is a target of GM604
Efficacy biomarkers	Plasma Total Tau	↓Reduced significantly at week 6 (28% below baseline, p=0.0369) ^2^	↑Increased	Statistically significant reduction
Target/efficacy biomarkers	Plasma TDP-43	↓Reduced significantly at week 12 (34% below baseline, p=0.0078) ^3^	↑Increased (6%)	Statistically significant reduction. Indicates GM6 has a neuroprotective effect and targeted and lowered TDP-43 levels
	CSF Cystatin C	↑Increased	↓Reduced	Indicates GM6 has a neuroprotective effect by increasing Cystatin C
Prognostic biomarkers	CSF pNFH	↓Reduced	↓Reduced, but to a lesser extent than in GM6 treated patients.	Higher pNFH is an indicator of higher disease progression rate.

We observed suggestive trends but no statistically significant changes in CSF biomarker levels ( Table 1). SOD1 levels decreased at week 6 (visit 7) following treatment with GM604 but increased following placebo treatment ^30^. Total CSF tau was decreased after end of week 2 (visit 6, final dose) of active treatment with GM604, whereas tau increased following placebo treatment ^26^. Cystatin C was increased after end of week 2 (visit 6, final dose) and week 12 (visit 8) following treatment with GM604, and was decreased following placebo treatment ^27, 28^.

Figure 1 compares CSF and plasma SOD1 levels at baseline (visit 1) and at the end of week 2 (visit 6, final dose) in the GM604 treated and placebo group. In Figure 1A and 1B, each point represents a single ALS patient, such that patients below the diagonal exhibit decreased SOD1 at visit 6 compared to visit 1. There was a trend towards decreased SOD1 in the CSF, but it was not statistically significant (p=0.123; one-tailed t-test; Figure 1A, Dataset 1 ^34^, Dataset 4 ^40^). For plasma measurements, however, SOD1 abundance was significantly lower at visit 6 compared to visit 1 (p=0.009, paired one-tailed t-test; Figure 1B)

Figure 2 shows the percentage change in slope by treatment interaction of plasma TDP-43 over time, from baseline (visit 1) through to week 12 (visit 8). The mean change in slope for the GM604 treated group was -3.513 pg/ml, which represents a 34% decrease, while the mean change in slope for the placebo group was 0.493 pg/ml, which represents a 6% increase (p=0.0078 for the difference between the slopes, -34% vs 6%, GM604 vs. placebo). To analyze disease progression, the results of the biomarker assays were analyzed using a mixed model repeated measures analysis. Commensurate with the design of the study, a mixed effects model was used to examine differences in the percentage change from baseline over time for each of the biomarkers. The covariance structure was used to model the intra-subject correlation. Since the percentage change from baseline is zero for all subjects at baseline, the y-intercept was removed from the model which forces the y-intercept to be 0. The explanatory variables that were added to the model include the week (2, 6, 12) as a numerical variable, treatment (GM604, placebo) and the treatment by week interaction. The model was run using all results through to week 6 and then again using all results through to week 12 separately. The p-value indicates a significant difference in slopes between GM604 and placebo up to week 12 ( Dataset 15 ^39^). 

Figure 3 shows percentage change in plasma total tau over time, from baseline (visit 1) through to week 6 (visit 7). The mean percentage change from baseline for plasma total tau in GM604 treated patients was -27.69%, while the mean percentage change from baseline for the placebo group was 13.23% (p = 0.0369, -27.69% vs 13.23%, Wilcoxon Rank Sum Test. Dataset 14 ^37^).

***Efficacy assessments***

**TUG, grip strength and HHD scores**. For weeks 2, 6 and 12, no significant treatment difference was observed between placebo and GM604 treatment groups with respect to TUG, grip strength and HHD scores ^18, 19^.

**ALSFRS-R**. Rates of change in ALSFRS-R are usually linear for any one individual patient (without any intervention), but are highly variable among different patients, ranging from rapid (1 year) to slow (>10 years) ^41^. Thus, to be able to measure any change in disease progression before and after treatment, ALSFRS-R was analyzed using mixed model analysis. The model allowed for differences in slopes before and after treatment in an attempt to observe disease modification. The slope for ALSFRS-R for the placebo group changed minimally before and after treatment, going from 0.037/day to -0.034/day. The slope for the GM604 group changed noticeably but not significantly before and after treatment, going from -0.046/day before treatment to -0.032/day after treatment. It appeared that the GM604 group had slowing of disease progression compared to pre-treatment ( Dataset 16 ^42^). At week 12, there was no statistically significant difference in ALSFRS-R between GM604- and placebo-treated groups ( Dataset 10 ^43^).

Outcomes were also compared to baseline features of placebo-treated definite ALS patients from recent clinical trials by NEALS ^29, 30^. In our GM604-treated patients, the monthly rate of decline per 30 days was -1.047 (I. The rate of decline per 30 days among historical controls was significantly greater (-1.97 per month; p = 0.0047, -1.047/mo vs -1.97/mo, mixed model, Dataset 17 ^44^), indicating improvement in GM604-treated patients compared to an independent historical control cohort.

**Forced vital capacity (FVC)**. At week 12, the total number of placebo- and GM604- treated patients was 4 and 7, respectively (one patient was excluded from week 12 assessments, see above). There was no statistically significant difference in the change of FVC from baseline between subjects who received placebo and those who received GM604 at week 12 ( Table 2, -11.5 vs -4.7, p=0.5393, two sample t-test, Dataset 11 ^45^).

**Table 2.** Mean baseline and week 12 FVC (% predicted) in all GALS-001 patients. N = number of patients. ^1^P-values were calculated by two-sample t Test. ^2^The P-value was obtained from a Wilcoxon Rank Sum Test. ( Dataset 11).

Time Point	Placebo	GM604
Baseline		
N	4	7
Mean FVC % Predicted	81.3	91.1
Week 12	4	7
Mean FVC % Predicted	69.8	86.4
Change from Baseline		
N	4	7
Mean FVC % Predicted change	-11.5	-4.7
P-values ^1^ Two sample t Test		0.5393
P-values ^2^ Wilcoxon Rank Sum Test		>0.9999

There were two sites included in this study ( Table 3). The screening visit and baseline assessment were separated by approximately 2 weeks. Intra-site variability was quite small for the placebo group at Site 001 and at both sites for the GM604 group (ranging from 0.3 to 3.0) and not statistically significant. While some variability between visits is expected, the drop of 15 points between screening visit and baseline assessment at Site 002 for the placebo group appeared very different than what was seen at the other site ( Table 3).

**Table 3.** Mean screening and baseline FVC (% predicted) of GALS-001 patients, separated by treatment type and site.

Time Point	Placebo All	Placebo Site 001	Placebo Site 002	GM604 All	GM604 Site 001	GM604 Site 002
Screening	87.30	70.50	104.00	90.40	89.80	91.00
Baseline	81.30	73.50	89.00	89.10	89.50	88.75

Only at Site 001 was there a statistically significant difference between placebo and GM604 treated group when using FVC data from baseline to week 12 ( Table 4, -28 vs -4.8, p=0.0268, two sample t-test ).

**Table 4.** Comparing change in FVC (% predicted) from baseline to week 12 at Site 001 GALS-001 patients between placebo and GM604 treated groups. ^1^P-values were calculated by two-sample t Test. ^2^The P-value was obtained from a Wilcoxon Rank Sum Test.

Time Point	Site 001 Placebo	Site 001 GM604
Baseline		
N	2	4
Mean	73.5	89.5
Week 12 N		
N	2	4
Mean	45.5	84.8
Change from Baseline N		
N	2	4
Mean	-28	-4.7
^1^P-values Two- sample t Test		0.0268
^2^P-values Wilcoxon Rank Sum Test		0.1052

### ALS Protocol GALS-C

The GALS-001 trial was under the restrictive inclusion criteria of definite ALS onset within 24 months and FVC >65%. As a follow-on study to investigate how an advanced ALS patient would respond to GM604, a single compassionate patient case study under protocol GALS-C outside of the restrictive inclusion criteria was initiated.

The patient was a 46-year old male diagnosed 10 years previously, quadriplegic for over eight years and on a ventilator. The patient received GM604 treatment in an identical dosing regimen as in GALS-001. The patient was too advanced to perform any of the clinical endpoint assessments such as ALSFRS-R, FVC etc. as in GALS-001, but personal clinical observations were recorded according to the patient’s condition.

Clinical observations revealed small but beneficial improvements from baseline to week 12. At week 2, the patient showed clearer articulation compared to the baseline assessment. At week 4, the patient's swallow volume had increased by 150%–200%. Oral fluid consumption reported by the patient was improved, measuring 250cc total without leakage. Mouth suction, as measured by water column height, increased from 5–8 cm to 10–15 cm with both 1/8 and 1/4 inch drinking straws. Speech, swallowing, and suction were used as primary metrics, based upon the rationale that the relatively short motor neurons in the tongue and lips would show improvements first.

In this advanced patient, CSF biomarkers SOD1, Cystatin C and total tau were all below the normal range at baseline. After 2 weeks of treatment with GM604 in this advance patient, all 3 biomarkers were upregulated towards their normal range (SOD1: 50–200 ng/ml; Cystatin C: 3.0–8.0 μg/ml; total tau: 100–350 pg/ml; see Table 5). In contrast, patients treated in this Phase 2A GALS-001 trial, diagnosed within 2 years of disease onset, had CSF biomarkers SOD1 and total tau at the high end of the normal range at the start of the trial, and at week 2, both of these biomarkers were downregulated towards their normal range. Cystatin C showed values that were at the low end of the normal range at the start of the trial and were up regulated towards their normal range by week 2. Table 5 represents a compilation summary of biomarker changes in patients after GM604 treatment in the GALS-001 and GALS-C trials.

**Table 5.** GALS-001 and GALS-C CSF biomarker results. GALS-C = Single compassionate patient treatment; GALS-T = GALS-001 treated group, and GALS-P = GALS-001 placebo group. ↑ = upregulation, ↓ = downregulation, DM* = disease modification, DP** = disease progression.

CSF Biomarkers	SOD1 GALS-C (N=1)	SOD1 GALS -T (N=8)	SOD1 GALS-P (N=4)	Cystatin C GALS-C (N=1)	Cystatin C GALS-T (N=8)	Cystatin C GALS - P (N=4)	total Tau GALS-C (N=1)	total Tau GALS-T (N=8)	total Tau GALS -P (N=4)
Sample-ID	Con.ng/ml	Con.ng/ml	Con.ng/ml	Conc.µg/ml	Conc.µg/ml	Conc.µg/ml	Con.pg/ml	Con.pg/ml	Con.pg/ml
**healthy range -** **CSF**	**50–200**	**50–200**	**50–200**	**3.0–8.0**	**3.0–8.0**	**3.0–8.0**	**100–350**	**100–350**	**100–350**
Baseline-CSF	27.228	186.6	137.94	1.97	3.11	3.23	60.55	305.03	386.85
standard deviation		168.3	56.39		1.35	0.78		122.3	182.93
Visit 6 (Week 2) -CSF	30.996	153.17	175.86	2.35	3.15	3.06	63.33	303.58	412.96
standard deviation		76.14	84.56		1.41	0.76		139.37	196.62
mean % Change V6-BL	13.84%	-3.75%	30.45%	19.29%	1.57%	-4.57%	4.59%	-1.16%	6.43%
standard deviation		26.20%	56.90%		8.49%	12.10%		15.79%	6.36%
**Comments**	**below** **range,** ↑=DM*	**high end** **of range,** ↓=DM*	**high end** **of range,** ↑=DP**	**below** **range,** ↑=DM*	**low end of** **range,** ↑=DM*	**low end of** **range,** ↓=DP**	**below** **range,** ↑=DM*	**high end** **of range,** ↓=DM*	**above** **range,** ↑=DP**

Plasma SOD1 measurements (GALS-001) Click here for additional data file. Copyright: © 2017 Kindy M et al. 2017 Data associated with the article are available under the terms of the Creative Commons Zero "No rights reserved" data waiver (CC0 1.0 Public domain dedication).

Plasma total tau measurements (GALS-001) Click here for additional data file. Copyright: © 2017 Kindy M et al. 2017 Data associated with the article are available under the terms of the Creative Commons Zero "No rights reserved" data waiver (CC0 1.0 Public domain dedication).

Plasma TDP-43 measurements (GALS-001) Click here for additional data file. Copyright: © 2017 Kindy M et al. 2017 Data associated with the article are available under the terms of the Creative Commons Zero "No rights reserved" data waiver (CC0 1.0 Public domain dedication).

CSF SOD1 measurements (GALS-001) Click here for additional data file. Copyright: © 2017 Kindy M et al. 2017 Data associated with the article are available under the terms of the Creative Commons Zero "No rights reserved" data waiver (CC0 1.0 Public domain dedication).

CSF total tau measurements (GALS-001) Click here for additional data file. Copyright: © 2017 Kindy M et al. 2017 Data associated with the article are available under the terms of the Creative Commons Zero "No rights reserved" data waiver (CC0 1.0 Public domain dedication).

CSF Cystatin C measurements (GALS-001) Click here for additional data file. Copyright: © 2017 Kindy M et al. 2017 Data associated with the article are available under the terms of the Creative Commons Zero "No rights reserved" data waiver (CC0 1.0 Public domain dedication).

CSF pNFH measurements (GALS-001) Click here for additional data file. Copyright: © 2017 Kindy M et al. 2017 Data associated with the article are available under the terms of the Creative Commons Zero "No rights reserved" data waiver (CC0 1.0 Public domain dedication).

Adverse events data (GALS-001) Click here for additional data file. Copyright: © 2017 Kindy M et al. 2017 Data associated with the article are available under the terms of the Creative Commons Zero "No rights reserved" data waiver (CC0 1.0 Public domain dedication).

Serious adverse event data (GALS-001) Click here for additional data file. Copyright: © 2017 Kindy M et al. 2017 Data associated with the article are available under the terms of the Creative Commons Zero "No rights reserved" data waiver (CC0 1.0 Public domain dedication).

ALS Functional Rating Scale – Revised (ALSFRS-R) data (GALS-001) Click here for additional data file. Copyright: © 2017 Kindy M et al. 2017 Data associated with the article are available under the terms of the Creative Commons Zero "No rights reserved" data waiver (CC0 1.0 Public domain dedication).

FVC data (GALS-001) Click here for additional data file. Copyright: © 2017 Kindy M et al. 2017 Data associated with the article are available under the terms of the Creative Commons Zero "No rights reserved" data waiver (CC0 1.0 Public domain dedication).

Biomarker data for GALS-C Click here for additional data file. Copyright: © 2017 Kindy M et al. 2017 Data associated with the article are available under the terms of the Creative Commons Zero "No rights reserved" data waiver (CC0 1.0 Public domain dedication).

Source table for calculating the percentage change from baseline to week 2 for plasma SOD1 Click here for additional data file. Copyright: © 2017 Kindy M et al. 2017 Data associated with the article are available under the terms of the Creative Commons Zero "No rights reserved" data waiver (CC0 1.0 Public domain dedication).

Source table for calculating the percentage change from baseline to week 6 for plasma total tau Click here for additional data file. Copyright: © 2017 Kindy M et al. 2017 Data associated with the article are available under the terms of the Creative Commons Zero "No rights reserved" data waiver (CC0 1.0 Public domain dedication).

Comparison of disease progression determined by changes in plasma TDP-43 Click here for additional data file. Copyright: © 2017 Kindy M et al. 2017 Data associated with the article are available under the terms of the Creative Commons Zero "No rights reserved" data waiver (CC0 1.0 Public domain dedication).

Source table for ALSFRS-R before and after treatment (GALS-001) Click here for additional data file. Copyright: © 2017 Kindy M et al. 2017 Data associated with the article are available under the terms of the Creative Commons Zero "No rights reserved" data waiver (CC0 1.0 Public domain dedication).

Source table comparing ALSFRS-R data in GM604 treated patients with data from the historical control cohort ^46, 47^ Click here for additional data file. Copyright: © 2017 Kindy M et al. 2017 Data associated with the article are available under the terms of the Creative Commons Zero "No rights reserved" data waiver (CC0 1.0 Public domain dedication).

## Discussion

### ALS Protocol GALS-001

This GALS-001 Phase 2A, multi-center, randomized, double-blind, placebo-controlled, pilot trial was performed as part of the development program for GM604. The study was designed to test proof of principle, with the objectives of testing the safety, tolerability and efficacy of GM604 in a small cohort of ALS patients, based upon changes in ALS biomarkers and measures of clinical progression ^29^.

Our findings show that GM604 is safe and tolerable at the doses administered in this study (i.e., 320 mg by IV bolus injection 3X/week for two consecutive weeks). Ad hoc analysis revealed that the GM604-treated group demonstrated improvements in disease outcomes, achieving statistical significance in FVC clinical data at week 12 at Site 001. GM604 also changed the expression levels of three ALS plasma biomarkers (SOD1, total tau, and TDP-43). The GM604-treated group exhibited a trend towards slower disease progression compared to placebo-treated patients. Although ALSFRS-R at week 12 did not show a statistically significant difference between the GM604-treated group and placebo patients, in ad hoc analysis there were trends for improvements.

Previous clinical studies in patients with ALS have suggested that biomarker concentrations in plasma, serum, and CSF can be predictive of disease progression ^32, 33^. Therefore, a primary endpoint of the present study was to examine the percentage change of each biomarker between baseline and week 12. Although changes in CSF biomarker levels were observed over time, from baseline through to week 12, no statistically significant changes were observed in CSF biomarkers SOD1, total tau, Cystatin C, and pNFH ^15– 19, 26– 28^. Plasma biomarkers, in contrast, showed stronger differences between GM604-treated and placebo-treated patients. For example, plasma TDP-43 was reduced significantly by 34% below baseline at week 12 ( Figure 2). Consistent with this, the slope in plasma TDP-43 from baseline to week 12 in GM604 treated patients (-3.513 pg/mL/wk which represent a change of -34%) was lower than that in placebo patients (0.493 pg/mL/wk which represent a change of 6%) (p = 0.0078, -34% vs 6%, mixed model; Figure 2). Plasma SOD1 in the GM604-treated group also showed a significant reduction at week 2 when compared with the placebo group (p = 0.009; one tailed t-test Figure 1B). Finally, plasma total tau reduction achieved statistical significance in percentage change at week 6 between the treated and placebo patients (p = 0.0369 95% CI -27.69% vs 13.23%, Wilcoxon Rank Sum Test, Dataset 14, Figure 3).

The biomarker results in GALS-001 suggests that GM6 modulates ALS disease through multiple pathways. Our findings suggest a tentative mechanism of action (MOA) by which GM6 could prolong motor neuron survival in ALS patients. We propose a “tripartite mechanism” ^8^. First, by reducing *SOD1* expression, GM6 may block accumulation of pathologic SOD1 aggregates in motor neurons. Second, by reducing mitochondrial gene expression and potentially mitochondrial abundance (decreasing total tau), GM6 may disrupt the mitochondrial (intrinsic) apoptotic pathway. Third, GM6 appears to activate developmental/mitotic pathways (Cystatin C), which may promote cellular repair, axonogenesis, and neuron projection.

We did not observe significant changes with respect to some clinical efficacy measures (HHD, TUG, grip strength). Early changes in muscle strength are difficult to measure accurately by HHD because the accuracy of HHD decreases with higher muscle strength ^19^. Grip strength and HHD assessments had great variability due to the different handedness of the patients along with the disease potentially affecting one side of the body in a slightly different manner than the other side. TUG may also not be an ideal clinical measurement for ALS trials because as ALS progresses, many patients with ALS are unable to perform TUG. In this trial, 50% of the patients receiving placebo treatment were not able to perform TUG at Week 12.

### ALS Protocol GALS-C

The GALS-C patient is an unusual case, having survived 10 years when the average life expectancy is 2 to 5 years ( http://www.alsa.org/about-als/facts-you-should-know.html). The GALS-C patient’s SOD1 and total tau biomarkers were below the normal range and GM604 upregulated them towards the normal range; whereas SOD1 and total tau biomarkers of GALS-001 trial patients were above normal range and GM604 downregulated them towards normal range. While it is difficult to establish strong conclusions from a single patient, these results suggest that GM604 may have homeostatic effects on biomarker abundance (i.e., decreasing biomarkers when abnormally elevated and increasing biomarkers when abnormally repressed). In this respect, GM604 may not strictly act as an agonist or antagonist, but may instead have more complex and patient-specific effects depending on baseline status. Further studies and analyses of larger patient cohorts will be needed to address this possibility.

For some analyses, patients in the present study were compared to placebo-treated patients from a clinical study designed to evaluate the safety and efficacy of ceftriaxone treatment in definite ALS patients ( Dataset 17 ^44^) ^46, 47^. The use of historical placebo data may increase the clinical relevance of efficacy and safety information that can be gleaned from the current trial ^21, 48^. This may reduce type I error and improve statistical power for evaluating outcomes and endpoints in a small study ^49^. However, when comparing these groups there are inherent variables between study populations that may lead to potential differences. For example, diagnostic criteria, the population with the disease, and concomitant standards of care can all lead to potential differences. The comparison with historical placebo data therefore needs to be interpreted with caution.

All data reported here have been submitted to the FDA. FDA has since encouraged Genervon to conduct a Phase 3 study under special protocol assessment process. Genervon is planning for the phase 3 clinical trial in 2017.

## Consent

Written informed consent for participation in the trial and publication of patient information was obtained from each patient.

## Data availability

The data referenced by this article are under copyright with the following copyright statement: Copyright: © 2017 Kindy M et al.

Data associated with the article are available under the terms of the Creative Commons Zero "No rights reserved" data waiver (CC0 1.0 Public domain dedication). 

Dataset 1. Plasma SOD1 measurements (GALS-001).

DOI, 10.5256/f1000research.10519.d153298 ^34^

Dataset 2. Plasma total tau measurements (GALS-001)

DOI, 10.5256/f1000research.10519.d153299 ^36^

Dataset 3. Plasma TDP-43 measurements (GALS-001)

DOI, 10.5256/f1000research.10519.d153300 ^38^

Dataset 4. CSF SOD1 measurements (GALS-001)

DOI, 10.5256/f1000research.10519.d153301 ^50^

Dataset 5. CSF total tau measurements (GALS-001)

DOI, 10.5256/f1000research.10519.d153302 ^40^

Dataset 6. CSF Cystatin C measurements (GALS-001)

DOI, 10.5256/f1000research.10519.d153303 ^51^

Dataset 7. CSF pNFH measurements (GALS-001)

DOI, 10.5256/f1000research.10519.d153304 ^52^

Dataset 8. Adverse events data (GALS-001)

DOI, 10.5256/f1000research.10519.d153305 ^53^

Dataset 9. Serious adverse event data (GALS-001)

DOI, 10.5256/f1000research.10519.d153306 ^54^

Dataset 10. ALS Functional Rating Scale – Revised (ALSFRS-R) data (GALS-001)

DOI, 10.5256/f1000research.10519.d153307 ^43^

Dataset 11. FVC data (GALS-001)

DOI, 10.5256/f1000research.10519.d153308 ^45^

Dataset 12. Biomarker data for GALS-C

DOI, 10.5256/f1000research.10519.d153309 ^55^

Dataset 13. Source table for calculating the percentage change from baseline to week 2 for plasma SOD1

DOI, 10.5256/f1000research.10519.d153310 ^35^

Dataset 14. Source table for calculating the percentage change from baseline to week 6 for plasma total tau

DOI, 10.5256/f1000research.10519.d153311 ^37^

Dataset 15. Comparison of disease progression determined by changes in plasma TDP-43

DOI, 10.5256/f1000research.10519.d153312 ^39^

Dataset 16. Source table for ALSFRS-R before and after treatment (GALS-001)

DOI, 10.5256/f1000research.10519.d153313 ^42^

Dataset 17. Source table comparing ALSFRS-R data in GM604 treated patients with data from the historical control cohort ^46, 47^.

DOI, 10.5256/f1000research.10519.d153314 ^44^

## Supplementary material

**Supplementary file 1: Article manuscript showing where each component of the CONSORT checklist has been adhered to.** Clinical trials must adhere to the CONSORT reporting guidelines.

**Supplementary file 2: Completed CONSORT flow diagram.** Clinical trials must adhere to the CONSORT reporting guidelines.
