# Supplementary material for: A Phase 2A randomized, double-blind, placebo-controlled pilot trial of GM604 in patients with Amyotrophic Lateral Sclerosis (ALS Protocol GALS-001) and a single compassionate patient treatment (Protocol GALS-C)
Source: F1000Res. 2017 Mar 7;6:230. [Version 1] doi: 10.12688/f1000research.10519.1 (PMC6051227; doi:10.12688/f1000research.10519.1)
Supplement: Supplementary file 18 [file f1000research-6-11337-s0017.tgz › f1afdd46-a8b9-484b-9aa3-1fcb8d369852.docx]

C1a

A Phase 2A randomized, double-blind, placebo-controlled pilot trial of GM604 in patients with Amyotrophic Lateral Sclerosis (ALS Protocol GALS-001) and a single compassionate patient treatment (Protocol GALS-C)

Mark S. Kindy[1], Paul Lupinacci[2], Raymond MW Chau[3], Tony Shum[3], Dorothy Ko[3*]

[1] Department of Pharmaceutical Sciences, College of Pharmacy, University of South Florida, Tampa, FL and the James A. Haley VAMC, Tampa, FL, USA.

[2] Department of Mathematics and Statistics, Villanova University, Villanova, PA, USA.

[3] Genervon Pharmaceuticals LLC, Pasadena, CA, USA.

*Corresponding author

Abstract

Amyotrophic lateral sclerosis (ALS) is a fatal neurodegenerative disease that lacks effective treatment options since its discovery 150 years ago. Genervon has discovered and developed GM604 (GM6) the endogenous fetal stage Insulin Receptor tyrosine kinase binding motoneuronotrophic factor regulator of the nervous system as a potential ALS therapy.

This was a 2-centers Phase 2A, randomized, double-blind, placebo-controlled pilot trial with 12 definite ALS patients diagnosed within 2 years of disease onset. Patients received 6 doses of GM604 or placebo, administered as slow IV bolus injections (320 mg) three times per week for 2 consecutive weeks. Objectives were to assess the safety and efficacy of GM604 based on ALSFRS-R, FVC and selected biomarkers (TDP-43, Tau and SOD1, pNFH). This report also includes results of compassionate treatment protocol GALS-C for an advanced ALS patient.

C1b

Definite ALS patients were randomized at each site to four GM604 treated and two placebos treated. 2 of 8 GM604-treated patients exhibited mild rash, but otherwise adverse event frequency was similar in treated and placebo groups. GM604 slowed functional decline (ALSFRS-R) when compared to a historical control (P = 0.005). At one study site, a statistically significant difference between treatment and control groups was found when comparing changes in respiratory function (FVC) between baseline and week 12 (P = 0.027). GM604 decreased plasma levels of key ALS biomarkers relative to the placebo group (TDP-43, P = 0.008; Tau, P = 0.037; SOD1, P = 0.009). The advanced ALS patient in compassionate treatment demonstrated improved speech, oral fluid consumption, mouth suction with GM604 treatment and biomarker improvements.

We observed favorable shifts in ALS biomarkers and improved functional measures during the Phase 2A study as well as in an advanced ALS patient. These data support GM604 as a therapeutic drug for ALS.

Introduction

Amyotrophic lateral sclerosis (ALS) is a devastating disease for which no effective treatment has been discovered[1]. During the last twenty years, dozens of ALS drug candidates have been tested but have unfortunately failed during clinical trials[2]. This astounding record of uniform failure may be attributed to the fact that the classic drug development model – which aims to design single-target drugs – is simply inadequate for rapid, complex and multifactorial diseases like ALS[3].

Genervon decided to look for and discovered endogenous regulators of the developing nervous system, and hypothesized that such regulators may have the capacity to monitor and repair neurological diseases[4,5]. Genervon's approach was to base drug design on these regulatory proteins, leading to development of GM604 (GM6)[6]. GM604 is a peptide with a sequence identical to one of the active sites of human motoneuronotrophic factor (MNTF)[7]. MNTF is an endogenous human embryonic stage neural regulatory and signaling peptide that controls the development, monitoring and correction of the human nervous system[4,5]. This activity of MNTF is replicated by GM604 to provide a potent disease-modifying drug candidate that modulates many processes including inflammation, apoptosis, and hypoxia[4,5,7]. In pre-clinical studies, we have shown that GM604 acts as a neuro-protective agent in animal models of neurological disease[7]. In these studies, GM604 was found to promote neuroprotection, neurogenesis, neural development, neuronal signaling, neural transport, and other processes[4-7]. Recently, we have demonstrated that GM604 modulates many ALS-associated genes, promoting decreased expression of superoxide dismutase (SOD1), repression of genes associated with the intrinsic apoptosis pathway, and increased expression of genes associated with mitosis and cell division[8].

C2a

C2b

This paper reports findings from a multi-center Phase 2A, double-blind, randomized, placebo-controlled pilot trial in 12 patients with Familial or Sporadic ALS diagnosed as definite ALS according to the El Escorial Criteria[9,10]. Objectives of the trial were to assess proof of principle; i.e., to determine whether a 2-week IV bolus treatment with GM604 can (i) be safely used and tolerated without significant adverse effects, (ii) favorably alter ALS biomarkers, and (iii) delay progression based upon key clinical indices. This report also includes results of protocol GALS-C for an advanced ALS patient who has been quadriplegic and on a ventilator since 2008 (IND number 120052).

Methods

C2a

C3a

C2a

This was a multi-center Phase 2A, double-blind, randomized, placebo-controlled pilot trial in 12 patients with Familial or Sporadic ALS. Objectives were to test the safety, tolerability and efficacy of GM604 and to assess changes in clinical disease progression and selected ALS biomarkers. GM604 has received Orphan Drug Designation 14-4247 by the FDA Office of Orphan Products Development for treatment of ALS and Orphan Designation (EU/3/16/1662) from the European Medical Commission. Genervon received Fast Track Designation for GM604 to treat ALS (IND number 118,420) by FDA Office of Drug Evaluation I, CDER. Genervon also received Fast Track designation for GM604 to treat Ischemic Stroke (IND number 77,789).

C3a

This report also includes results of protocol GALS-C for an advanced ALS patient who has been quadriplegic and on a ventilator since 2008 (IND number 120052). It is an Expanded Access Use applied by a physician to treat his/her individual patient. The physician submits a new IND request with Form 1571 to FDA including treatment protocol, CV, IRB approval, Informed Consent Form, Medical License etc. and a Letter of Authorization (LOA) signed by the sponsor to refer to the sponsor's IND for information regarding the investigational drug in Investigator's Brochure, Chemistry, Manufacturing and

C23, C24

Controls (CMC) information, and pharmacology and toxicology. After FDA approves the Expanded Access Treatment request by the physician, an IND number 120052 is assigned for the Expanded Access Use for the GALS-C patient treatment with GM60404. GM60404 is only shipped to the physician after the physician received FDA's Study May Proceed letter. All components required by FDA are fulfilled before FDA will assign an IND number and allow the treatment to proceed. Since GALS-C is not a clinical trial, it is not registered with clinicaltrials.gov. FDA now has a simpler form for Individual Patient Expanded Access Applications (FDA Form 3926).

Randomization

7a, 8a, 8b,
9, 10, 11a

Patients who qualified for the study were enrolled and assigned a unique patient number. The patient's initials and identification number were written on all source documents. Only the site number and patient's study ID number were written on CRF pages, documents sent to central readers, and CSF and blood samples sent to central lab for processing.

Patients fulfilling the eligibility criteria were assigned randomization codes, starting with number 0101, with 0100 series for Site 001 and 0200 series for Site 2. The patient number was assigned in sequential order as the patient enrolled. 6 patients were enrolled at each site. 8 patients were randomized to receive GM604 and 4 patients were randomized to receive placebo control. The statistical analysis team generated a list of randomization code and sent the list to the pharmacist of each site. Study site pharmacist retained the original treatment randomization schedule in a secure location. All activities of this study were conducted in a double-blinded, randomized, placebo controlled manner.

Ethics statement

The Phase 2A study was performed in compliance with the current International Conference on Harmonization (ICH) Good Clinical Practice (GCP) guidance and the current version of the Declaration of Helsinki of the World Medical Association[11]. The final protocol and informed

C23,
C24

C23, C24

consent form were reviewed and approved by the Columbia University Institutional Review Board (CU IRB) for Site 001 (Columbia University Medical Center) and by the Partners Human Research Committee (PHRC) for Site 002 (Massachusetts General Hospital). All patients who participated were fully informed about the study in accordance with GCP guidelines, federal regulations, HIPAA, and local requirements[12]. The trial was posted on clinicaltrials.gov on May 8, 2013. (NCT01854294)[13]. The GALS-C is not a clinical trial but an Expanded Access for compassionate treatment. IRB approval was received from Bay Area Regional IRB of Dignity Health.

ALS Protocol GALS-001.

Subject Population

C4a C4b

There are a total of two study sites: Columbia University Medical Center, New York and Massachusetts General Hospital. Definite ALS patients were randomized at each site to four GM604 treated and two placebo treated. Eligible patients met the El Escorial criteria for ALS[9,10]. At screening, symptom onset had occurred within the previous 24 months and forced vital capacity (FVC) was ≥65% of predicted capacity based upon age, height, and gender. Mean disease duration was 8.15 months, ranging from 2.7 to 16.5 months across treatment groups. Patients in the placebo group reported a slightly longer duration of disease, with a median duration of 8.90 months, compared with a median of 5.24 months for patients in the GM604 treatment group. The demographic profile of the placebo and GM604 treatment groups was matched in terms of age, with medians of 54.5 and 56.0 years in the placebo and treatment groups, respectively. The mean age of patients was 55.7 years, ranging from 45 to 68 across treatment groups. The majority of patients (66.7%; 8/12) were male. Gender distribution was slightly different in the two treatment groups, with an equal number of males and females in the

C13, C14, C15

placebo group (2/2) and a majority of males in the GM604 treatment group (75%; 6/8). All 4 of the females were at least 2 years post-menopausal. All 12 patients were Caucasian.

Patients were excluded if they had a bleeding disorder, allergy to local anesthetics, or medical or surgical conditions in which lumbar puncture was contraindicated, e.g., elevated cerebrospinal fluid (CSF) pressure. Prohibited medications included anti-platelet or anticoagulant drugs such as Plavix, non-steroidal anti-inflammatory drugs (NSAIDs), ticlid, warfarin or coumadin. Patients may have been on a stable dose of riluzole for at least a month before screening, but riluzole was not initiated during the trial. We note that some biomarker data were missing due to hemolysis of samples, technical issues, or patients who missed clinical appointments for sample collection. These missing data were excluded from analyses.

Procedures

Following screening, patients were randomized to receive GM604 (n=8) or placebo (n=4). Patients received 6 doses of 320 mg GM604 or placebo, administered as slow IV bolus injections on Monday, Wednesday, and Friday of weeks 1 and 2. Clinical assessments included the ALS Functional Rating Scale – Revised (ALSFRS-R) [19], FVC[20,21,22], timed up & go (TUG)[23], and hand-held dynamometry (HHD)[24]. Assessements were conducted at screening, before the first dose (baseline), after the last (6th) dose at week 2, and at weeks 6 and 12. Safety and tolerability were evaluated based on the frequency of adverse events, vital signs, electrocardiography (ECG) measurements, physical and neurological examinations, safety laboratory monitoring, and hypersensitivity and injection site reactions[15]. The following visit windows were allowed: visits 1 (baseline and first dosing) to 6 (last dosing, 2 weeks): ± 1 day; visit 7 (4 weeks after last dosing, 6 weeks total): ± 7 days; visit 8 (10 weeks after last dosing, 12 weeks total): ± 14 days. We note that one patient (in the GM604 treatment group) returned to

Germany where he resides and did not return for the week 12 assessment, although he did contact investigators to provide ALSFRS-R by phone. A total of 11 patients thus received all 6 doses of the study drug, with one patient receiving 5 doses of the drug.

Biomarkers

The biomarkers SOD1, phosphorylated neurofilament heavy chain (pNFH)[37], total tau, and TDP-43 were assessed at baseline, after the initial week 2 (4th) dose (plasma only), after the last (6th) dose (also in week 2) and at weeks 6 and 12. TDP-43 (TAR DNA-binding protein 43, transactive response DNA binding protein 43 kDa) is a protein encoded by the *TARDBP* gene. Mutations in the *TARDBP* gene are associated with neurodegenerative disorders including ALS. [14,15,16, 17,18] We note that some biomarker data were missing due to technical issues with sample processing, or patients who missed clinical appointments for sample collection. These missing data were excluded from analyses.

Efficacy assessments

The ALSFRS-R is used to assess disability in ALS patients. It is a total score derived from sub-scores in the following categories: speech, salivation, swallowing, handwriting, cutting food, dressing and hygiene, turning in bed, walking, climbing stairs, dyspnea, orthopnea, and respiratory insufficiency. The score decreases as the disease progresses[19].

The FVC, measured as a percentage, is used to assess respiratory function and is an indicator of disease progression. FVC also decreases with disease progression[20,21,22].

TUG is used to predict falls in ALS. In this study, TUG in ambulatory participants with no assistance was measured and recorded with videotaping[23] The TUG was measured in seconds rounded to 1 decimal place, with smaller estimates indicating that a patient can walk faster. As

ALS progresses, however, the walking pace may slow, or the patient may be unable to perform TUG. In the present study, TUG performed with assistance was excluded and treated as missing data.

C6a

HHD is used to measure muscle strength. HHD measures are dependent on the ability of the evaluator to overpower the subject's strength[24]. In this study, the clinician stabilized the limb segment while encouraging the patient to exert as much force as possible against an isometric HHD, and the maximum force was recorded by the HHD. Each site was tested in duplicate (triplicate if the first 2 results were more than 15% apart) and the result was measured in pounds using 1 decimal. The average of replicates for each clinical site was calculated and used in the analysis for each of the time points.

C12a, C12b

Statistical Analyses

The percentage change from baseline of each biomarker in plasma and CSF was compared between treatments using a 2-sample t-test and Wilcoxon Rank Sum test. Progressive changes in clinical endpoints were examined using mixed effects modeling (ALSFRS-R, FVC, TUG, grip strength and HHD scores). Rates of disease progression were compared between GM604- and placebo-treated patients. Additionally, we made comparisons to placebo-treated patients from the Northeast ALS Consortium (NEALS) database showing stable rates of decline (https://www.alsconsortium.org/).

Results

ALS Protocol GALS-001

14a

Study Initiation date was 16 May 2013 (first Subject pre-screened0, 03 September 2013 (first Subject screened), Study completion/Termination Date (last Subject completed) was 11 April 2014.

Safety

C19

Of 12 patients enrolled in the study, 9 reported at least one adverse event. Overall, in the GM604 treatment group, 5 of 8 patients experienced at least one treatment emergent adverse event (TEAE) and 4 of 4 patients in the placebo group experienced at least 1 TEAE. No unexpected findings were observed. Consistent with protocol-defined expected adverse reactions, the most frequently reported AEs by GM604-treated patients in the present study were falls (4 patients, 50%), puncture site pain (3 patients, 37.5%), rash (2 patients) and headache (2 patients, 25%). Of these most commonly reported TEAEs in GM604-treated patients, falls (1 patient, 25%), puncture site pain (1 patient, 25%) and headache (2 patients, 50%) were reported in placebo-treated patients.

Adverse events in the 'general disorders and administration site conditions' system organ class (SOC) were the most frequently experienced adverse events (7 patients and 61 total events in both the GM604 and placebo-treated groups). A serious adverse event that required inpatient hospitalization, shortness of breath 24 days after the first dose of GM604 (12 days after the last dose), was experienced by one patient in the GM604 treatment group. This patient received the full 6 doses of GM60404 treatment and then left the study site and flew back

to Germany. There was no additional GM604 administered to this patient during the hospital stay in Germany that could have affected the outcome of the results.

It was determined by investigators that this serious adverse event was most likely due to the natural progression of ALS and was thus unrelated to the investigational product. No deaths or withdrawals due to adverse events occurred.

There were no clinically meaningful differences noted between patients who received GM604 and those who received placebo for changes over time in clinical laboratory tests, hematology parameters, or urinalysis results. There were no clinically meaningful differences noted between patients who received GM604 and those who received placebo for changes over time in ECGs, vital signs, physical findings, neurological examination, or other observations related to safety.

Grade 1 hypersensitivity reactions were reported by one patient receiving placebo (visit 2 during week 1) and one patient receiving GM604 (visit 5 during week 2). All other patients reported an absence of hypersensitivity (Grade 0) reactions. There was no indication of QT prolongation as no patient receiving GM604 had a QT or QTcB (QT corrected using Bazett's formula) result above 450 msec.

Biomarker findings

Previous clinical studies in patients with ALS have suggested that biomarker concentrations in plasma, serum, and CSF can be predictive of disease progression[25-32]. Therefore, a primary endpoint of the present study was to examine the percentage change of each biomarker between baseline and week 12.

In plasma samples, percentage change in plasma SOD1 at visit 6 (end of week 2) was lower than at baseline (p=0.0550, two sample t-test) following GM604 treatment compared with placebo which did not lower SOD1 (Table 1, Figure 1, Dataset 1 and Dataset 13). Percentage change in plasma total tau was significantly decreased, approximately -28% below baseline (p=0.0369 95% CI, Wilcoxon Rank Sum test) at week 6 (visit 7) after active GM604 treatment compared to placebo (Table 1, Figure 3, Dataset 2 and Dataset 14). Percentage change in slope by treatment interaction in plasma TDP-43 from baseline (visit 1) through to week 12 (visit 8) was -34% in the GM604 treated group and +6% in the placebo group (p=00078 95% CI). The p-value of 0.0078 indicates the significance of the difference in slopes between GM604 and placebo (Table 1, Figure 2, Dataset 3 and Dataset 15) up to week 12.

C15, C16, C17, C18

We observed suggestive trends but no statistically significant changes in CSF biomarker levels (Table 1). SOD1 levels decreased at week 6 (visit 7) following treatment with GM604 but increased following placebo treatment[29]. Total CSF tau was decreased after end of week 2 (visit 6, final dose) of active treatment with GM604, whereas tau increased following placebo treatment[25]. Cystatin C was increased after end of week 2 (visit 6, final dose) and week 12 (visit 8) following treatment with GM604, and was decreased following placebo treatment[26,27]. GM604

Figure 1 compares CSF and plasma SOD1 levels at baseline (visit 1) and at the end of week 2 (visit 6, final dose) in the GM604 treated and placebo group. In Figure 1A and 1B, each point represents a single ALS patient, such that patients below the diagonal exhibit decreased SOD1 at visit 6 compared to visit 1. There was a trend towards decreased SOD1 in the CSF, but it was not statistically significant (p=0.123; one-tailed t-test; Figure 1A, Dataset 1, Dataset 4).

For plasma measurements, however, SOD1 abundance was significantly lower at visit 6 compared to visit 1 (p=0.009, paired one-tailed t-test; Figure 1B)

Figure 2 shows the percentage change in slope by treatment interaction of plasma TDP-43 over time, from baseline (visit 1) through to week 12 (visit 8). The mean change in slope for the GM604 treated group was -3.513 pg/ml, which represents a 34% decrease, while the mean change in slope for the placebo group was 0.493 pg/ml, which represents a 6% increase (p=0.0078 95% CI for the difference between the slopes, -34% vs 6% GM604 vs. placebo). To analyze disease progression, the results of the biomarker assays were analyzed using a mixed model repeated measures analysis. Commensurate with the design of the study, a mixed effects model was used to examine differences in the percentage change from baseline over time for each of the biomarkers. The unstructured covariance structure was used to model the intra-subject correlation. Since the percentage change from baseline is zero for all subjects at baseline, the y-intercept was removed from the model which forces the y-intercept to be 0. The explanatory variables that were added to the model include the week (2, 6, 12) as a numerical variable, treatment (GM604, placebo) and the treatment by week interaction. The model was run using all results through to week 6 and then again using all results through to week 12 separately. The p-value indicates the significance of the difference in slopes between GM604 and placebo (Dataset 15) up to week 12.

C17, C18

Figure 3 shows percentage change in plasma total tau over time, from baseline (visit 1) through to week 6 (visit 7). The mean percentage change from baseline for plasma total tau in GM604 treated patients was -27.69%, while the mean percentage change from baseline for the placebo group was 13.23% (GM604p = 0.0369 95% CI -27.69% vs 13.23% Wilcoxon Rank Sum Test. Dataset 14).

Efficacy assessments

TUG, grip strength and HHD scores

For weeks 2, 6 and 12, no significant treatment difference was observed between placebo and GM604 treatment groups with respect to TUG, grip strength and HHD scores[23,24].

ALSFRS-R

C15, C16, C17, C18

Rates of change in ALSFRS-R are usually linear for any one individual patient (without any intervention), but are highly variable among different patients, ranging from rapid (1 year) to slow (>10 years)[33]. Thus, to be able to measure any change in disease progression before and after treatment, ALSFRS-R was analyzed using mixed model analysis. The model allowed for differences in slopes before and after treatment in an attempt to observe disease modification. The slope for ALSFRS-R for the placebo group changed minimally before and after treatment, going from -0.037/day to -0.034/day. The slope for the GM604 group changed noticeably but not significantly before and after treatment, going from -0.046/day before treatment to -0.032/day after treatment. It appeared that the GM604 group had slowing of disease progression compared to pre-treatment (Dataset 16). At week 12, there was no statistically significant difference in ALSFRS-R between GM604- and placebo-treated groups.(Dataset 10)

Outcomes were also compared to baseline features of placebo-treated definite ALS patients from recent clinical trials by NEALS[28,29]. In our GM604-treated patients, the monthly rate of decline per 30 days was -1.047 (I. The rate of decline per 30 days among historical controls was significantly greater (-1.97 per month; p = 0.0047 95% C -1.047/mo vs -1.97/mo, mixed model, Dataset 17), indicating improvement in GM604-treated patients compared to an independent historical control cohort.

C15, C16, C17, C18

Forced vital capacity (FVC)

At week 12, the total number of placebo- and GM604- treated patients was 4 and 7, respectively (one patient was excluded from week 12 assessments, see above). There was no statistically significant difference in the change of FVC from baseline between subjects who received GM604 and those who received placebo at week 12 (Table 2, -11.5 vs -4.7, p=0.5393, two sample t-test, Dataset 11).

There were two sites included in this study (Table 3). The screening visit and baseline assessment were separated by approximately 2 weeks. Intra-site variability was quite small for the placebo group at Site 001 and at both sites for the GM604 group (ranging from 0.3 to 3.0) and not statistically significantGM604. While some variability between visits is expected, the drop of 15 points between screening visit and baseline assessment at Site 002 for the placebo group appeared very different than what was seen at the other site (Table 3).

C18

Only at Site 001 was there a statistically significant difference between GM604 treated group and placebo when using FVC data from baseline to week 12 (Table 4, -28 vs -4.8, p=0.0268, two sample t-test).

ALS Protocol GALS-C

C2b

The GALS-001 trial was under the restrictive inclusion criteria of definite ALS onset within 24 months and FVC >65%. As a follow-on study to investigate how an advanced ALS patient would respond to GM604, a single compassionate patient case study under protocol GALS-C outside of the restrictive inclusion criteria was initiated.

The patient was a 46-year old male diagnosed 10 years previously, quadriplegic for

C3a

over eight years and on a ventilator. The patient received GM604 treatment in an identical dosing regimen as in GALS-001. The patient was too advanced to perform any of the clinical endpoint assessments such as ALSFRS-R, FVC etc. as in GALS-001, but personal clinical observations were recorded according to the patient’s condition.

C17, C18, C20, C21, C22

Clinical observations revealed small but beneficial improvements from baseline to week 12. At week 2, the patient showed clearer articulation compared to the baseline assessment. At week 4, the patient's swallow volume had increased by 150%-200%. Oral fluid consumption reported by the patient was improved, measuring 250cc total without leakage. Mouth suction, as measured by water column height, increased from 5-8 cm to 10-15 cm with both 1/8 and 1/4 inch drinking straws. Speech, swallowing, and suction were used as primary metrics, based upon the rationale that the relatively short motor neurons in the tongue and lips would show improvements first.

C17, C18, C20, C21, C22

In this advanced patient, CSF biomarkers SOD1, Cystatin C and total tau were all below the normal range at baseline. After 2 weeks of treatment with GM604 in this advance patient, all 3 biomarkers were upregulated towards their normal range (SOD1: 50-200 ng/ml; Cystatin C: 3.0-8.0 □g/ml; total tau: 100-350 pg/ml; see Table 5). In contrast, patients treated in this Phase 2A GALS-001 trial, diagnosed within 2 years of disease onset, had CSF biomarkers SOD1 and total tau at the high end of the normal range at the start of the trial, and at week 2, both of these biomarkers were downregulated towards their normal range. Cystatin C showed values that were at the low end of the normal range at the start of the trial and were up regulated towards their normal range by week 2. Table 5 represents a compilation summary of biomarker changes in patients after GM604 treatment in the GALS-001 and GALS-C trials.

Discussion

ALS Protocol GALS-001

The GALS-001 Phase 2A, multi-center, randomized, double-blind, placebo-controlled, pilot trial was performed as part of the development program for GM604. The study was designed to test proof of principle, with the objectives of testing the safety, tolerability and efficacy of GM604 in a small cohort of ALS patients, based upon changes in ALS biomarkers and measures of clinical progression[28].

Our findings show that GM604 is safe and tolerable at the doses administered in this study (i.e., 320 mg by IV bolus injection 3X/week for two consecutive weeks). Ad hoc analysis revealed that the GM604-treated group demonstrated improvements in disease outcomes, achieving statistical significance in FVC clinical data at week 12 at Site 001. GM604 also changes the expression levels of three ALS plasma biomarkers (SOD1, total tau, and TDP-43). The GM604-treated group exhibited a trend towards slower disease progression compared to placebo-treated patients. Although ALSFRS-R at week 12 did not show a statistically significant difference between the GM604-treated group and placebo patients, in ad hoc analysis there were trends for improvements.

Previous clinical studies in patients with ALS have suggested that biomarker concentrations in plasma, serum, and CSF can be predictive of disease progression[31,32]. Therefore, a primary endpoint of the present study was to examine the percentage change of each biomarker between baseline and week 12. Although changes in CSF biomarker levels were observed over time, from baseline through to week 12, no statistically significant changes

were observed in CSF biomarkers SOD1, total tau, Cystatin C, and pNFH[20-27]. Plasma biomarkers, in contrast, showed stronger differences between GM604-treated and placebo-treated patients. For example, plasma TDP-43 was reduced significantly by 34% below baseline at week 12 (Figure 2). Consistent with this, the slope in plasma TDP-43from baseline to week 12 in GM604-treated patients (-3.513 pg/mL/wk which represent a change of -34%) was lower than that in placebo patients (0.493 pg/mL/wk which represent a change of 6%) (p = 0.0078 CI 95% -34% vs6% Mixed model; Figure 2). Plasma SOD1 in the GM604-treated group also showed a significant reduction at week 2 when compared with the placebo group (p = 0.009; one tailed t-test Figure 1B). Finally, plasma total tau reduction achieved statistical significance in percentage change at week 6 between the treated and placebo patients (p = 0.0369 95% CI -27.69% vs 13.23% Wilcoxon Rank Sum Test, Dataset 14, Figure 3).

The biomarker results in GALS-001 suggests that GM6 modulates ALS disease through multiple pathways. Our findings suggest a tentative mechanism of action (MOA) by which GM6 could prolong motor neuron survival in ALS patients. We propose a “tripartate mechanism”. First, by reducing *SOD1* expression, GM6 may block accumulation of pathologic SOD1 aggregates in motor neurons. Second, by reducing mitochondrial gene expression and potentially mitochondrial abundance (decreasing total tau), GM6 may disrupt the mitochondrial (intrinsic) apoptotic pathway. Third, GM6 appears to activate developmental/mitotic pathways (Cystatin C), which may promote cellular repair, axonogenesis, and neuron projection.

We did not observe significant changes with respect to some clinical efficacy measures (HHD, TUG, grip strength). Early changes in muscle strength are difficult to measure accurately by HHD because the accuracy of HHD decreases with higher muscle strength[24]. Grip strength

C20, C21, C22

and HHD assessments had great variability due to the different handedness of the patients along with the disease potentially affecting one side of the body in a slightly different manner than the other side. TUG may also not be an ideal clinical measurement for ALS trials because as ALS progresses, many patients with ALS are unable to perform TUG. In this trial, 50% of the patients receiving placebo treatment were not able to perform TUG at Week 12.

ALS Protocol GALS-C

The GALS-C patient is an unusual case, having survived 10 years when the average life expectancy is 2 to 5 years (http://www.alsa.org/about-als/facts-you-should-know.html). The GALS-C patient's SOD1 and total tau biomarkers were below the normal range and GM604 upregulated them towards the normal range; whereas SOD1 and total tau biomarkers of GALS-001 trial patients were above normal range and GM604 downregulated them towards normal range. While it is difficult to establish strong conclusions from a single patient, these results suggest that GM604 may have homeostatic effects on biomarker abundance (i.e., decreasing biomarkers when abnormally elevated and increasing biomarkers when abnormally repressed). In this respect, GM604 may not strictly act as an agonist or antagonist, but may instead have more complex and patient-specific effects depending on baseline status. Further studies and analyses of larger patient cohorts will be needed to address this possibility.

C20, C21, C22

For some analyses, patients in the present study were compared to placebo-treated patients from a clinical study designed to evaluate the safety and efficacy of ceftriaxone treatment in definite ALS patients (Dataset 17) [34,35]. The use of historical placebo data may increase the clinical relevance of efficacy and safety information that can be gleaned from the current trial[36,37]. This may reduce type I error and improve statistical power for evaluating outcomes and endpoints in a small study[38]. However, when comparing these groups there are

C20, C21, C22

inherent variables between study populations that may lead to potential differences. For example, diagnostic criteria, the population with the disease, and concomitant standards of care can all lead to potential differences. The comparison with historical placebo data therefore needs to be interpreted with caution.

All data reported here have been submitted to the FDA. FDA has since encouraged Genervon to conduct a Phase 3 study under special protocol assessment process. Genervon is planning for the phase 3 clinical trial in 2017.

Consent

C4a, C23, C24

Written informed consent for participation in the trial and publication of patient information was obtained from each patient.

Data availability

C21

17 Datasets are submitted to F1000Research

Author contributions

DK designed the study. MK prepared the first draft of the manuscript. PL, RMWC and TS contributed to manuscript preparation. All authors were involved in the revision of the draft manuscript and have agreed to the final content.

Competing interests

Dorothy Ko is an executive of the company and has ownership interest in Genervon Biopharmaceuticals, LLC, the sponsor of this trial.

Grant information

C25

This study was funded by Genervon Biopharmaceuticals, LLC.

Acknowledgments

C25

Editorial assistance was provided by WCCT Global, LLC, funded by Genervon Pharmaceuticals, LLC.

References

1. DeLoach A, Cozart M, Kiaei A, Kiaei M. A retrospective review of the progress in amyotrophic lateral sclerosis drug discovery over the last decade and a look at the latest strategies. Expert Opin Drug Discov. 2015;10(10):1099-118.
2. Katz JS, Barohn RJ, Dimachkie MM, Mitsumoto H. The Dilemma of the Clinical Trialist in Amyotrophic Lateral Sclerosis: The Hurdles to Finding a Cure. Neurol Clin. 2015;33(4):937-47.
3. Eisen E, Amyotrophic Lateral Sclerosis is a Multifactorial Disease. Muscle & Nerve 1995;18:741-752.
4. Chau R, Wu X, Zhao L, Ren F, Jan J, Neuronotrophic factor. Chin J Neuroanat. 1990;6:129-138.
5. Chau R, Ren F, Huang W, Jen L. Muscle neurotrophic factors specific for anterior horn motoneurons of rat spinal cord. Recent Adv Cell Mol Biol. 1992;5:89-94.
6. Lu H, Le WD, Xie YY, Wang XP. Current Therapy of Drugs in Amyotrophic Lateral Sclerosis. Curr Neuropharmacol. 2016;14(4):314-21.
7. Yu J, Zhu H, Ko D, Kindy MS. Motoneuronotrophic factor analog GM604 reduces infarct volume and behavioral deficits following transient ischemia in the mouse. Brain Res. 2008;1238:143-53.
8. Swindell WR, Bojanowski K, Kindy M *et al.* GM60404 down-regulates SOD1 and alters expression of 89 genes associated with amyotrophic lateral sclerosis [v1; not peer reviewed]. *F1000Research* 2016, **5**:2836 (poster) (doi: 10.7490/f1000research.1113511.1).
9. GM604GM604

BR Brooks. El Escorial World Federation of Neurology criteria for the diagnosis of amyotrophic lateral sclerosis. Subcommittee on Motor Neuron Diseases/Amyotrophic Lateral Sclerosis of the World Federation of Neurology Research Group on Neuromuscular Diseases and the El Escorial "Clinical limits of amyotrophic lateral sclerosis" workshop contributors. J Neurol Sci. 1994;124:96–107.

10. BR Brooks, RG Miller, M Swash, TL Munsat, the World Federation of Neurology Research Group on Motor Neuron Diseases. El Escorial revisited: revised criteria for the diagnosis of amyotrophic lateral sclerosis. Amyotroph Lateral Scler Other Motor Neuron Disord. 2000;1:293–299.
11. http://www.ich.org/home.html
12. Nagata E, Ogino M, Iwamoto K, Kitagawa Y, Iwasaki Y, Yoshii F, Ikeda JE; ALS Consortium Investigators. Bromocriptine Mesylate Attenuates Amyotrophic Lateral Sclerosis: A Phase 2a, Randomized, Double-Blind, Placebo-Controlled Research in Japanese Patients. PLoS One. 2016;11(2):e0149509.
13. ClinicalTrials.gov registry Identifier: NCT01854294. Genervon Biopharmaceuticals, LLC. GM60404 Phase 2A Randomization Double-blind Placebo Controlled Pilot Trial in Amyotrophic Lateral Disease (ALS) (GALS-001) 8 May 2013.
14. Kasai T, Tokuda T, Ishigami N, Sasayama H, Foulds P, Mitchell DJ, Mann DM, Allsop D, Nakagawa M. "Increased TDP-43 Proein in Cerebrospinal Fluid of Patients with Amyotrophic Lateral Sclerosis." *Acta Neuropathol* 117.1 (2009 Jan;117(1):55-62. doi: 10.1007/s00401-008-0456-1.): 55-62.

15. Noto Y, Shibuya K, Sato Y, Kanai K, Misawa S, Sawai S, Mori M, Uchiyama T, Isose S, Nasu S, Sekiguchi Y, Fujimaki Y, Kasai T, Tokuda T, Nakagawa M, Kuwabara S. "Elevated CSF TDP-43 levels in amyotrophic lateral sclerosis: specificity, sensitivity, and a possible prognostic value." *Amyotroph Lateral Scler.* 12.2 (2011): 140-3.

16. Egawa N, Kitaoka S, Tsukita K, Naitoh M, Takahashi K, Yamamoto T, Adachi F, Kondo T, Okita K, Asaka I, Aoi T, Watanabe A, Yamada Y, Morizane A, Takahashi J, Ayaki T, Ito H, Yoshikawa K, Yamawaki S, Suzuki S, Watanabe D, Hioki H, Kaneko T, Makioka K, Okam. "Drug screening for ALS using patient-specific induced pluripotent stem cells." *Sci Transl Med.* 4.145 (2012): 145ra104.

17. Ling SC, Polymenidou M, Cleveland DW. "Converging mechanisms in ALS and FTD: disrupted RNA and protein homeostasis." *Neuron.* 79.3 (2013): 416-38

18. Ling JP, Pletnikova O, Troncoso JC, Wong PC. "TDP-43 repression of nonconserved cryptic exons is compromised in ALS-FTD." *Science* 349.6248 (2015): 650-5

19. Cedarbaum JM, Stambler N, Malta E, Fuller C, Hilt D, Thurmond B, Nakanishi A. The ALSFRS-R: a revised ALS functional rating scale that incorporates assessments of respiratory function. BDNF ALS Study Group (Phase III). J Neurol Sci. 1999;169:13-21.

20. Traynor BJ, Zhang H, Shefner JM, Schoenfeld D, Cudkowicz ME; NEALS Consortium. Functional outcome measures as clinical trial endpoints in ALS. Neurology. 2004;63(10):1933-5.

21. Lunetta C, Lizio A, Sansone VA, Cellotto NM, Maestri E, Bettinelli M, Gatti V, Melazzini MG, Meola G, Corbo M. Strictly monitored exercise programs reduce motor deterioration in ALS: preliminary results of a randomized controlled trial. J Neurol. 2016;263(1):52-60.

22. Ruiz-López FJ, Guardiola J, Izura V, Gómez-Espuch J, Iniesta F, Blanquer M, López-San Román J, Saez V, De Mingo P, Martínez S, Moraleda JM. Breathing pattern in a phase I clinical trial of intraspinal injection of autologous bone marrow mononuclear cells in patients with amyotrophic lateral sclerosis. Respir Physiol Neurobiol. 2016;221:54-8.

23. Montes J1, Cheng B, Diamond B, Doorish C, Mitsumoto H, Gordon PH. The Timed Up and Go test: predicting falls in ALS. Amyotroph Lateral Scler. 2007;8(5):292-5.

24. Beck M, Giess R, Wurffel W, Magnus T, Ochs G, Toyka KV. Comparison of maximal voluntary isometric contraction and drachman's hand-held dynamometry in evaluating patients with ALS. Muscle & Nerve. 1999;9:1265-1270.

25Pijnenburg YA, Verwey NA, van der Flier WM, Scheltens P, Teunissen CE. Discriminative and prognostic potential of cerebrospinal fluid phosphoTau/tau ratio and neurofilaments for frontotemporal dementia subtypes. Alzheimers Dement (Amst). 2015;1(4):505-12.

26. Ren Y, Zhu W, Cui F, Yang F, Chen Z, Ling L, Huang X. Measurement of cystatin C levels in the cerebrospinal fluid of patients with amyotrophic lateral sclerosis. Int J Clin Exp Pathol. 2015;8(5):5419-26.

27Wilson ME, Boumaza I, Lacomis D, Bowser R. Cystatin C: a candidate biomarker for amyotrophic lateral sclerosis. PLoS One. 2010;5(12):e15133.

28. Pasinetti GM, Ungar LH, Lange DJ, Yemul S, Deng H, Yuan X, Brown RH, Cudkowicz ME, Newhall K, Peskind E, Marcus S, Ho L. Identification of potential CSF biomarkers in ALS. Neurology. 2006;66(8):1218-22.24.
29. Winer L, Srinivasan D, Chun S, Lacomis D, Jaffa M, Fagan A, Holtzman DM, Wancewicz E, Bennett CF, Bowser R, Cudkowicz M, Miller TM. SOD1 in cerebral spinal fluid as a pharmacodynamic marker for antisense oligonucleotide therapy. JAMA Neurol. 2013;70(2):201-7.
30. Ganesalingam J, An J, Shaw CE, Shaw G, Lacomis D, Bowser R. Combination of neurofilament heavy chain and complement C3 as CSF biomarkers for ALS. J Neurochem. 2011;117(3):528-37.
31. Ganesalingam J, An J, Bowser R, Andersen PM, Shaw CE. pNfH is a promising biomarker for ALS. Amyotroph Lateral Scler Frontotemporal Degener. 2013;14(2):146-9.
32. Oeckl P, Jardel C, Salachas F, Lamari F, Andersen PM, Bowser R, de Carvalho M, Costa J, van Damme P, Gray E, Grosskreutz J, Hernández-Barral M, Herukka SK, Huss A, Jeromin A, Kirby J, Kuzma-Kozakiewicz M, Amador Mdel, Mora JS, Morelli C, Muckova P, Petri S, Poesen K, Rhode H, Rikardsson AK, Robberecht W, Rodríguez Mahillo AI, Shaw P, Silani V, Steinacker P, Turner MR, Tüzün E, Yetimler B, Ludolph AC, Otto M. Multicenter validation of CSF neurofilaments as diagnostic biomarkers for ALS. Amyotroph Lateral Scler Frontotemporal Degener. 2016;17(5-6):404-13.
33. Ravits J. et al. ALS Motor Phenotype heterogeneity, focality and spread. Neurology 2009;73:805-811.
34. Berry JD, Shefner JM, Conwit R, Schoenfeld D, Keroack M, Felsenstein D, Krivickas L, David WS, Vriesendorp F, Pestronk A, Caress JB, Katz J, Simpson E, Rosenfeld J,

Pascuzzi R, Glass J, Rezania K, Rothstein JD, Greenblatt DJ, Cudkowicz ME; Northeast ALS Consortium. Design and initial results of a multi-phase randomized trial of ceftriaxone in amyotrophic lateral sclerosis. PLoS One. 2013;8(4):e61177.

35. Merit. E. Cudkowicz, et al. Safety and efficacy of ceftriaxone for amyotrophic lateral sclerosis: a multi-stage, randomised, double-blind, placebo-controlled trial. The Lancet Neurology. 2014;13(11):1083-1091.
36. Gordon et al for WALS Study Group. Efficacy of minocycline in patients with amyotrophic lateral sclerosis: a phase III randomized trial. Lancet Neurol 2007; 6:1045-53.
37. Boylan K et al. Phosphorylated neurofilament heavy subunit (pNF-H) in peripheral blood and CSF as a potential prognostic biomarker in amyotrophic lateral sclerosis J Neurol Neurosurg Psychiatry. 2013;84:4 467-472.
38. Use of historical control data for assessing treatment effects in clinical trials. Pharm Stat. 2014; Pharm Stat. 2014;13(1):41–54.
