# Supplementary material for: A Phase 2A randomized, double-blind, placebo-controlled pilot trial of GM604 in patients with Amyotrophic Lateral Sclerosis (ALS Protocol GALS-001) and a single compassionate patient treatment (Protocol GALS-C)
Source: F1000Res. 2017 Mar 7;6:230. [Version 1] doi: 10.12688/f1000research.10519.1 (PMC6051227; doi:10.12688/f1000research.10519.1)

CONSORT FLOW DIAGRAM

A Phase 2A randomized, double-blind, placebo-controlled pilot trial of GM604 in patients with Amyotrophic Lateral Sclerosis (ALS Protocol GALS-001) and a single compassionate patient treatment (Protocol GALS-C)

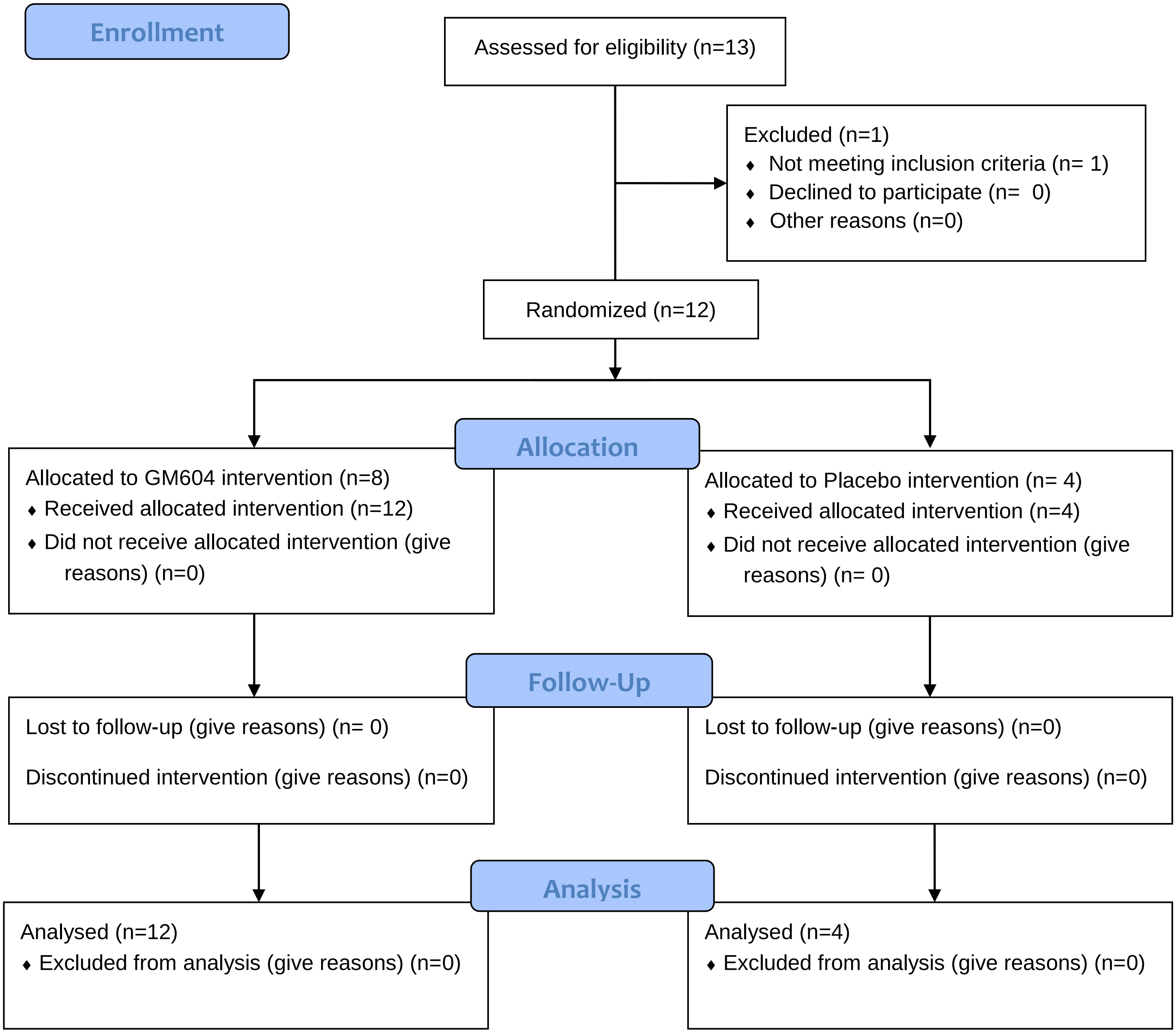

Supplement: Supplementary file 19 [file f1000research-6-11337-s0018.tgz › bce3e2bf-9a5a-43d7-937e-629b0d5465d2.pdf]
