# Supplementary material for: A Phase 2A randomized, double-blind, placebo-controlled pilot trial of GM604 in patients with Amyotrophic Lateral Sclerosis (ALS Protocol GALS-001) and a single compassionate patient treatment (Protocol GALS-C)
Source: F1000Res. 2017 Mar 7;6:230. [Version 1] doi: 10.12688/f1000research.10519.1 (PMC6051227; doi:10.12688/f1000research.10519.1)
Supplement: Peer review history

# Peer review: Referee response for version 1

**DOI:** 10.5256/f1000research.11337.r35982

**Published:** 2018-07-17

**Authors:** Héctor R. Martínez

**Affiliations:** 1 Institute of Neurology and Neurosurgery, Hospital Zambrano Hellion, Monterrey Institute of Technology and Higher Education (ITESM), San Pedro Garza García, Mexico

I am sending my comments and criticism to the research article entitled A Phase 2A randomized, double-blind, placebo-controlled pilot trial of GM604 in patients with Amyotrophic Lateral Sclerosis (ALS Protocol GALS-001) and a single compassionate patient treatment (Protocol GALS-C), written by Kindy M, Lupinacci P, Chau R, Shum T and Ko D

 This article describes that the GM604 is a peptide with a sequence identical to one of the active motoneuronotrophic factor. This peptide modulates many ALS-associated genes, promoting decreased expression of SOD1, repression of genes associated with the intrinsic apoptosis pathway, and increased expression of genes associated with mitosis and cell division. It is a very interesting peptide that may be investigated in this neurologic disorder as well as in other neurodegenerative and neurovascular disorder including Ischemic Stroke. 

1. GM604 according to this papers it is a safe drug, therefore I consider has to be applied in larger number ALS patients since this trial includes only 12 ALS patients treated with this peptide and 4 placebo treated patients
2. This article is described as multi-center phase 2A, double-blind, randomized, placebo-controlled pilot trial, however in the abstract is reported a 2-center trial
3. ALS diagnosed as definite ALS was performed according to the El Escorial Criteria. However, it is not described whether clinical and neurophysiologic criteria including Awaji criteria were used.
4. The procedures used for patient evaluation in this trial were performed with scientific rigor, However: 
  a)There is a small number of ALS patients (8 in treatment and 4 in placebo group)
  b) In this paper the authors describe objective measurements including ALSFRS-R Time Up and Go and Hand-Held Dynamometry and FVC, although most of these objective measurements are not very sensitive to follow up in research drug trial, however I consider these measurements have to be performed every 4 weeks at least during 24 weeks mostly the ALSFRS-R
5. The authors did not describe in the methods section the type of CSF analysis performed. However, they described in the section of statistical analysis that biomarker in plasma and CSF was compared between treatment groups
6. The biomarkers in blood and CSF are not considered as objective measurement of clinical outcome, probably some biomarkers may be used as possible effect of experimental drugs or dell therapy into the motor neuronal environment. The biomarkers so far reported in the literature are considered as some potential candidates for diagnosis or prognosis in ALS patients including Cystatin C, SOD1, pNfH, Neurofilament Heavy Chain Tau/tau ratio, and CSF Cytokine Pathway reported recently for our research group
7. A table reporting adverse events between ALS treated patients and controls has to be integrated in the paper.
8. This is a research article that involves treated and control ALS subjects therefore I believe that historical controls are not needed
9. The authors did not report in the methods, type of CSF analysis were done in treated and control ALS patients. However, in the statistical analysis they compared biomarker results in blood and CSF between treatments
10. All patients included in this research are in an early state of ALS. There is a short time between clinical onset and inclusion to the trial (8.9 and 5.2 months in treated patients and placebo group respectively). I believe that these patients could be followed at least for 6 months after inclusion to this research protocol
11. It was previously reported that the average decline in the ALSFRS-R score in ALS patients is -1.1 points per month and -13.32 points per year. Therefore, in this trial a comparison has to be done between treated ALS patients and those in the placebo group.
12. Merit Cudkowicz describe a decline in ALSFRS-R score of 3.6 point for placebo group and -2.2 in patients using dexpramipexole dose after three-months period follow-up. Since the observation period in this research trial (12 weeks) is similar than Cudkowicz report, a comparison of ALSFRS-R score among them is worthwhile
13. Recently in ALS patients, slow and fast progressors have been identified using a probabilistic model with the ALSFRS-R score over 12 months follow up in ALS patients under placebo treatment. I judge this analysis may be useful in this research protocol as well as in a future trial with GM 604
14. In this research article objective measurements were performed including ALSFRS-R, Time Up and Go and Hand-Held Dynamometry at screening, baseline, 2, 6 and 12 weeks as well as safety and tolerability that only was applied in eleven patients. However, in TUG, Grip Strength and HHD scores, no significant treatment differences were observed between placebo and treatment groups. No statistically significant changes were found in the FVC among ALS treated and placebo. The slope for ALSFRS-R for the placebo group changed minimally before and after treatment and in those patients treated with GM 604 had no statistically significant changes at 12 weeks. I believe this no representative changes among treated and placebo group is due to a) small number of patients and controls included in this trial 2) short period of follow up and 3) the objective measurements used in this trial need to be strengthened
15. The objective measurements in this trial could be: The Tractography including measuring of the Anisotropic Fraction of the pyramidal tract that is mostly originated in upper motor neurons of the frontal motor strip and the Motor Evoked Potential at baseline and 3 to 6 months after inclusion. Although these objective measurements may be more useful to objectively determine the potential protective effect of this peptide to motor neurons.

 

 Finally, I consider that the authors presented an interesting paper to be published as a short communication including some modification I have described above that include in future trials a higher number of patients as well as improve the objective measurement about safety and efficacy. To combine results obtained in this trial with some other (advanced ALS in compassionate treatment) it is no suggested since they already reported that results in other report.

I have read this submission. I believe that I have an appropriate level of expertise to confirm that it is of an acceptable scientific standard, however I have significant reservations, as outlined above.

# Peer review: Referee response for version 1

**DOI:** 10.5256/f1000research.11337.r29817

**Published:** 2018-03-12

**Authors:** Nitin Saksena

**Affiliations:** 1 Beijing Institute of Genomics, Chinese Academy of Sciences , Beijing, NSW, China; 2 China National GeneBank, Shenzhen, China

In this paper Kindy M *et al*., for Genervon have conducted a Phase 2A randomized, double blind, placebo-controlled pilot trial of GM604 in patients with Amyotrophic Lateral Sclerosis (ALS).

 Genervon discovered an endogenous embryonic stage regulator of the human nervous system named Motoneuronotrophic Factor (MNTF). GM604 is a peptide with a sequence identical to one of the active sites of human motoneuronotrophic factor (MNTF)7. MNTF is an endogenous human embryonic stage neural regulatory and signaling peptide that controls the development, monitoring and correction of the human nervous system. Genervon has taken advantage of these functional attributes of MNTF peptide, termed GM604, in this paper as a potential therapy for ALS. GM6 has been modeled upon an insulin receptor tyrosine kinase binding motoneuronotrophic factor within the developing central nervous system. GM604 modulates many ALS-associated genes, promoting decreased expression of superoxide dismutase (SOD1), repression of genes associated with the intrinsic apoptosis pathway, and increased expression of genes associated with mitosis and cell division. 

 This study relates to a 2-center phase 2A, randomized, double-blind, placebo-controlled pilot trial with 12 definite ALS patients who were diagnosed within 2 years of disease onset. During the trial, patients received 6 doses of GM604 or placebo, administered as slow IV bolus injections (3x/week, 2 consecutive weeks). The main objectives of this Phase 2A trial were to assess the safety and efficacy of GM604 based on ALSFRS-R, FVC and selected biomarkers (TDP-43, Tau and SOD1, pNFH). In addition, this clinical trial was also extended to a compassionate inclusion and treatment protocol GALS-C for an advanced ALS patient. Thus, this protocol includes patients with early onset of ALS and 1 advanced ALS patient.

- In this study, definite ALS patients were randomized to one of two treatment groups (GM604, n = 8; placebo, n = 4). During the trial, 2 of 8 GM604-treated patients exhibited mild rash, but otherwise adverse event frequency was similar in treated and placebo groups.
- GM604 slowed functional decline (ALSFRS-R) when compared to a historical control (P = 0.005). At one study site, a statistically significant difference between treatment and control groups was found when comparing changes in respiratory function (FVC) between baseline and week 12 (P = 0.027).
- GM604 decreased plasma levels of key ALS biomarkers relative to the placebo group (TDP-43, P = 0.008; Tau, P = 0.037; SOD1, P = 0.009). SOD-1 showed considerable reduction in plasma, with increased levels in placebo arm. Percentage change in plasma total tau was significantly decreased, approximately -28% below baseline (p=0.0369 95% at week 6 (visit 7) after active GM604 treatment compared to placebo. Percentage change in slope by treatment interaction in plasma TDP-43 from baseline (visit 1) through to week 12 (visit 8) was -34% in the GM604 treated group and +6% in the placebo group (p=00078 95% CI). The p-value of 0.0078 indicates a significant difference in slopes between GM604 and placebo up to week 12.
- The advanced ALS patient in compassionate treatment demonstrated improved speech, oral fluid consumption, mouth suction with GM604 treatment and biomarker improvements.

 

**Comments**: First, although the trial was small in nature with the inclusion of only 12 patients (8 in the treatment arm, and 4 in the placebo arm), it clearly demonstrates that the drug GM604 is completely safe to use, with only minor adverse events that could be clinically managed. The trial has been very well managed, scrutinized, clinically and experimentally planned/executed, along with thorough experimentation for the analysis of biomarkers. The statistical analysis within the realm of the study is to-the-point, and the statistical assessments of plasma and CSF-derived biomarker data between baseline and treatment time points is rigorous. Overall, there is also a considerable experimental rigor in the study, and provides an unbiased view point of safety, coupled with the modulation of gene expression in response to the drug. The procedures for measuring ALSFRS efficacy measurements that include FVC, TUG, HHD, etc. have been performed with clinical rigor.

 Second, the trial also clearly demonstrates that the genes involved in ALS, and the biomarkers of the disease (TDP-43, Tau, SOD-1) showed favourable expression shift upon treatment with GM604, suggesting the genes being its possible targets. This also shows the evidence that the GM604 was able to alter the expression of certain ALS genes and its biomarkers in the positive direction, an evidence not shown before with any drug in the market for ALS treatment.

 Although improved functional measurements during the Phase 2A study and in advanced ALS patient were observed, they failed to meet statistical significance. A larger trial is needed in the light of these findings. It is also understandable from the authors point of view that the clinical improvement measures in ALS are highly variable and difficult to achieve with a drug that is mechanistically different, acting on gene modulation. In addition, most drug trialled for ALS encounter the same problem, and the drug GM604 not only concurs with other drugs, but also is in line with the natural progression and history of the disease. One significant advantage GM604 offers is the multi-target effect, which none of the ALS drugs have achieved. Thus, it is likely that with repeated use of GM604, which modulates gene expression, patients will start showing improvement once the function of the impaired genes, involved in ALS, is restored to their normal function.

 In the CSF,

- there were suggestive trends with no statistically significant changes in CSF biomarker levels. SOD1 levels decreased at week 6 (visit 7) following treatment with GM604, but increased following placebo treatment.

- Total CSF tau was decreased after end of week 2 (visit 6, nal dose) of active treatment with GM604. Tau increased following placebo treatment.
- Cystatin C increased after end of week 2 (visit 6, nal dose) and week 12 (visit 8) following treatment with GM604, and was decreased following placebo treatment.

These are positive and very interesting indications at the gene expression of these highly ALS-related biomarkers. What other genes authors encountered alongside these biomarkers? This is key in unravelling functionally-related genes.

 At the level of gene analysis, I believe authors have done an excellent job with the key ALS genes in plasma and CSF compartments but more functional analysis of genes is needed that can guide us towards the genes that are mechanistically related to the ones authors have analysed, and the genes that fit in the bigger schema of neurodegeneration.

**Overall comments**: It is a tightly controlled study with logical set of clinical and experimental parameters. Interesting to note is the multi-target nature of GM604, which has not been shown by any other drug for ALS. Mechanistically, the drug is tentative but larger clinical trials can evaluate a more precise mechanism. The way it stands currently, it is clear that this neuromodulator acts on a large number of genes that are involved in neurodegeneration, therefore a clear definition of genes and pathways is needed to address its precise mechanism. It also appears likely its utility in other neurodegenerative diseases.

 The clinical improvement scores were minimal and transitory, but this is expected and is in line with clinical history of patients, duration of the study (12 weeks), and the nature of ALS disease as such. As the drug works on gene expression, more time is needed for improvement in clinical scores, along with repeated use.

 Overall, authors have provided an excellent discussion in relation to previously published studies and this study is unique and exhaustive in terms of assessments of various clinical and experimental parameters.

 All in all, a bigger study is warranted to conclude on statistical improvement in clinical parameters, in addition to involving more exhaustive panoply of genes that can critically demonstrate the relationship with biomarkers SOD-1, Tau, TDP-43 and Cystatin, etc. This is critical!

I have read this submission. I believe that I have an appropriate level of expertise to confirm that it is of an acceptable scientific standard.

# Peer review: Referee response for version 1

**DOI:** 10.5256/f1000research.11337.r24163

**Published:** 2017-07-13

**Authors:** Walter G. Bradley

**Affiliations:** 1 Department of Neurology, Miller School of Medicine, University of Miami, Miami, FL, USA

GM604 is a potentially an interesting molecule for the treatment of ALS. Therefore, the results of this Phase IIa trial need to be carefully reviewed. There have been many similar Phase IIa trials of potentially interesting drugs for ALS, which have subsequently failed in large Phase III trials for 3 main reasons: too small numbers; the variability of ALS leads to cohort effects [better patients entering one arm of the study will make the treatment in that arm appear more favorable]; over-enthusiastic interpretation of results of a preliminary Phase II trial.

 I am very surprised that clinical investigators from the 2 trial sites are not included in the authorship of the paper. At the least, I would like to see a reason, and letters from the heads of those clinical sections that did the study, Drs. Cudkowicz and Mitsumoto, stating that they have read the paper and support its contents and conclusions. Without this, the current paper is a sponsor-derived report.

 The authors use the term "multi-center", and this should be changed to "two-center".

 Regarding safety: The authors should provide the FVC values for the patient who experienced respiratory distress leading to hospitalization, in order to allow assessment of whether this SAE could have been due to the drug. They should also provide more information about the rash in one patient - type, distribution, severity, response to treatment, and any resultant change in the trial.

 Regarding efficacy: The trial showed no significant change in the clinical parameters studied. This is not surprising since the trial only lasted 12 weeks. The differences between FVC values in the 2 sites is probably related to variability of the disease, not to treatment effect. Since the study had a placebo arm, there is no reason to include reference to historical controls, and that section of the results should be deleted. The authors comment that "to be able to measure any change in disease progression before and after treatment, the ALSFRS-R was analyzed using mixed model analysis." However, since they had pre-screening values on only 3 of their 12 subjects, no statistical model can provide such an assessment. Therefore, I do not support the assertion that they were able to examine pre-treatment progression, and recommend deletion of the statement in the Abstract that [we] "observed .... improved functional measures."

 Regarding biomarkers: Most of this paper is devoted to changes in potential biomarkers. Though the changes are interesting and worthy of report, the editors and the readers should understand that these changes do not prove efficacy. The gold standard remains demonstrating efficacy in clinically relevant measures of disease.

 Regarding the compassionate use patient: Since no significant clinical effect was seen in the 8 patients receiving active treatment and followed for 12 weeks, it is difficult to treat as reliable the unblinded clinical observations reported. This case is of interest to the sponsors of the study, but should not be included in a scientific report of this Phase IIa trial.

I have read this submission. I believe that I have an appropriate level of expertise to confirm that it is of an acceptable scientific standard, however I have significant reservations, as outlined above.

# Response

**Published:** 2017-08-27

**Authors:** Mark Kindy

**Affiliations:** USF, USA

We appreciate Dr. Bradley’s review of 7/17/2017. The following are explanations to the questions in his review.

Efficacy: The 12 week, 8 treated and 4 placebo pilot trial is not expected by anyone to see any trend or result. Genervon was willing to do the proof of concept trial hoping that the unique endogenous regulator peptide therapy drug candidate GM6 can proof to be efficacious despite the obstacles of too small and too short a trial. Although a larger trial is needed to confirm these findings, the present data are encouraging and support GM604 as an ALS drug candidate. Genervon is planning a Phase 3 ALS trial under Special Protocol Assessment suggested by FDA in 2017 in the US. Enrollment detail will be announced later.

Biomarker: Biomarkers are not the gold standard but highly significant to proof not only MOA but modulation of critical corrupted disease specific gene/protein expression of treated ALS patients (not in vitro or in vivo studies). Not only Merit is intrigued she encourage us to move forward because of these unique data. Most Pharma are talking to us because of the biomarker data of the treated ALS patients.

Efficacy: The FDA approved for Genervon to use and show historical control due to only 4 placebo patients in pilot trial.

FVC data: For the patient who experienced respiratory distress leading to hospitalization, the information can be found in the Datasets. Dataset 11 listed all FVC data for each subject. Dataset 9 informed that Subject 203 had SAE. Dyspnea which is unrelated to GM604, had moderate respiratory disorders NEC, no breathing abnormalities, but had shortness of breath. Subject 203 lived in Germany and flew back to Germany soon after receiving 6 doses. The MGH PI determined that the shortness of breath is unrelated to GM604 in the SAE report. This SAE occurred in Germany on 5-Dec 2013 after the patient flew back from Boston to Germany, 14 days after he received his last dose (6 ^th^ dose) on 22-Nov-2013 in Boston. He did not complete any other assessment after Visit 6, except he gave ALSFRS-R by phone for Visit 12. He should not have been allowed to be enrolled since he was not planning to comply with the protocol requirement. Subject 203 FVC data are as follows: pre-screening 6-Aug-13 96%, screening 5-Nov-13 79%, Visit 1 (Baseline, dose 1) 11-Nov-13 75%, Visit 6 (Week 2, dose 6) 22-Nov-13 84%. Please note that his FVC decreased from screening (79%) to baseline (75%) before first dose. His FVC improved to 84% at Visit 6 after 6 doses compared to baseline and screening.

Rash: Dataset 8 listed all the AEs including rash. Subject 203 had rash on forehead and along crease from nose to mouth on 15 Nov 2013, 5 days from start of dosing, mild, not SAE, unlikely related to GM604 according to PI and was resolved completely. Subject 207 had rash below injection site in left arm on 6 Feb 2014 (visit 5), 11 days from 1 ^st^ dosing. Rash is local, not SAE, possibly related to GM604, moderate, resolved completely. Subject did not receive the last dose (Visit 6).

Statistical analysis: of ALSFRS-R pre-and post-treatment between GM604 treated and placebo, our statistician used the ALSFRS-R from ***screening ***to visit 1 (dose 1), not from ***pre-screening*** to visit 1. Screening was supposed to be within two weeks from first dose. The ALS Phase 2A Study start date was August 2013. Pre-screening data are not part of the study. The pre-screening data were taken from the subject's clinical record before study start as a guide for the PI to consider recruiting the patient, although pre-screening data were reported in Dataset 10. As you can see in Dataset 10, the pre-screening dates were two to three months before study start in August: Subject 102 had pre-screening on 6/24/2013, Subject 201 pre-screening was on 5/16/2013, Subject 202 has pre-screening on 6/4/2013. Therefore, there were data from all twelve subjects (not three) from ***screening*** to baseline to be used in the mixed model analysis for pre-treatment progression.

Inclusion of data from the compassionate use patient: The compassionate use patient had ALS for 10 years and is quadriplegic and is on ventilator full time. His ALS disease has progressed much more than any of the subjects in the trial. GM6 was discovered as an endogenous multi-target regulator based on our innovative hypothesis and paradigm. Based on many pre-clinical studies, Genervon has confidence in efficacy of GM6 and was willing to treat even very advance ALS patients under expanded access IND. Reporting the findings of the very advance ALS patient functional responses and his biomarkers response to GM604 is of interest as a footnote because most clinical trials would not have included patients at such advanced state.

We hope this addresses the concerns.
